# Synthesis of Novel Derivatives of 4,6-Diarylpyrimidines and Dihydro-Pyrimidin-4-one and *In Silico* Screening of Their Anticancer Activity

**DOI:** 10.2174/0115701794356958241024082646

**Published:** 2024-12-05

**Authors:** Oleksandr V. Onipko, Veronika Stoianova, Oleksandr V. Buravov, Valentyn A. Chebanov, Alexander Kyrychenko, Eugene S. Gladkov

**Affiliations:** 1 Enamine Ltd., 67 Winston Churchill St., Kyiv 02660, Ukraine;; 2 Institute of Chemistry and School of Chemistry, V. N. Karazin Kharkiv National University, 4 Svobody Sq., Kharkiv 61022, Ukraine;; 3 State Scientific Institution “Institute for Single Crystals”, Institute of Functional Materials Chemistry of National Academy of Sciences of Ukraine, 60 Nauky Ave., Kharkiv 61072, Ukraine

**Keywords:** Organic synthesis, heterocycles, α-aminoamidine, dihydropyrimidine, molecular docking, anticancer activity

## Abstract

Derivatives of pyrimidinone, dihydropyrimidinone, and 2,4-diaryl-substituted pyrimidines were synthesized by cyclocondensation of α-aminoamidines with various saturated carbonyl derivatives and their analogs. The therapeutic potential of the newly synthesized derivatives for cancer treatment was evaluated using molecular docking calculations. The molecular docking results indicate that some of the synthesized diaryl derivatives of pyrimidine exhibit high binding affinity towards PIK3γ.

**Aims and Objectives:**

4,6-Diaryl-substituted pyrimidines have shown high inhibitory potency against phosphoinositide 3-kinases (PI3Ks), which are important targets in oncology. Inhibition of PI3Ks could potentially be a viable therapy for human cancers.

**Materials and Methods:**

The synthesis of pyrimidinone and dihydropyrimidinone derivatives as well as a series of 2,4-diaryl-substituted pyrimidines were described. These compounds were synthesized by cyclocondensation of α-aminoamidines with various saturated carbonyl derivatives and their analogs.

**Results:**

Derivatives of pyrimidinone, dihydropyrimidinone, and 2,4-diaryl-substituted pyrimidines were synthesized by combining α-aminoamidines with various saturated carbonyl derivatives and their analogs. By adjusting the large substituents in the 2-position, we gained the ability to modify the therapeutic properties of the resulting compounds. The potential of the newly synthesized derivatives for cancer treatment was assessed using molecular docking calculations. The results of the docking calculations suggest that some of the synthesized diaryl derivatives of pyrimidine have a strong binding affinity towards PIK3γ, making them promising candidates for the development of new anticancer medications.

**Conclusion:**

We synthesized some pyrimidinones, dihydropyrimidinones, and 2,4-diaryl-substituted pyrimidines by combining α-aminoamidines with various saturated carbonyl derivatives and similar compounds. Molecular docking results suggest that certain diaryl derivatives of pyrimidine have a strong binding affinity for PIK3γ. Moreover, diphenyl derivatives of pyrimidine exhibited dual inhibitory activity against PI3K and tubulin, showing promise for the development of next-generation microtubule-targeting agents for use in combination therapies.

## 1 INTRODUCTION

Amidines are valuable reagents for many cyclocondensations and are commonly used in the synthesis of heterocyclic compounds. For example, Vidal *et al*. used amidines to synthesize pyrimidines [1]. Another application is the preparation of substituted 4-pyrimidinols by condensing various ethyl-β-ketoesters under ultrasonic activation with different amidine hydrochlorides [1]. A new, efficient method for synthesizing pyrimidines involves Cu-catalyzed and 4-HO-TEMPO-mediated [3 + 3] annulation of commercially available amidines with saturated ketones [2]. This approach uses a new protocol for the synthesis of pyrimidines through a cascade reaction of oxidative dehydrogenation/annulation/ oxidative aromatization *via* direct β-C(sp3)−H-functionalization of saturated ketones followed by annulation with amidines [2].

A straightforward and selective reaction involving ketones, aldehydes, or esters with amidines, along with TEMPO and a recyclable Fe(II) complex prepared *in situ*, produces a range of pyrimidine derivatives with a wide tolerance for different functional groups. The reactions likely occur through a sequence involving TEMPO complexation, enamine addition, transient α-occupation, β-TEMPO elimination, and cyclization [3].

In our previous work [4], we demonstrated that α-aminoamidines can serve as substrates for synthesizing imidazole- and pyrimidine-containing building blocks, which can then be further transformed [5]. Additionally, we recently showed that α-aminoamidines can be used to synthesize a new series of tetrahydroquinazolines by reacting them with bisbenzylidene cyclohexanones [6]. The *in situ* reaction of the generated enone with acetamidine resulted in methyl-substituted pyrimidine [7]. We also found that the NaOH-catalyzed rearrangement of propargylic hydroxylamines provides highly stereoselective access to Cbz-protected β-enaminones, and the subsequent synthesis of pyrimidines further demonstrates the synthetic potential of these β-enaminones [8].

A simple one-pot synthesis of trifluoromethylated pyrimidines has been proposed. This method involves the cyclocondensation of fluorinated aryl-2-bromenones with alkylamidines [9]. The pyrimidine core is formed through a cascade reaction involving Michael aza addition, intramolecular cyclization, and dehydrohalogenation/ dehydration. The reaction conditions are mild, and the selectivity is high, resulting in a high yield of the desired heterocycles. The unique impact of the trifluoromethyl group on the reaction pathway has been demonstrated.

A set of ligands based on a 2,6-di(pyrimidin-4-yl)pyridine framework was created, and their ability to form complexes with Zn(II) and Cu(II) was assessed using UV/vis spectroscopy in buffered aqueous solution HEPES [10]. This core was combined with elements of screening hits, resulting in highly potent, selective tankyrase inhibitors that act as novel three-pocket binders [11]. A series of substituted pyrimidines were synthesized in high yield by reacting 1-(4-bromophenyl)-3-thiazol-5-yl-prop-2-en-1-one with various derivatives of β-aminoamidines [12]. The compound was cyclized with three different amidine hydrochlorides as well as guanidine hydrochloride to yield the corresponding pyrimidine [13]. The key intermediates 5-benzyl-2-phenylpyrimidin-4(3H)-ones or (E)-5-benzylidene-2-phenyl- 5,6-dihydropyrimidin-4(3H)-ones were conveniently obtained by cyclization of the acetates of Baylis–Hillman adducts and benzamidine hydrochloride in the presence of sodium ethoxide at room temperature [14].

In our previous studies [4, 6], we have shown that α-aminoamidines can be easily used to synthesize various pyrimidine derivatives. By using their acetates as reagents in a pyridine solvent, we can produce cyclocondensation products even with low reactivity electrophilic reagents under mild conditions. Additionally, utilizing α-aminoamidines and unsaturated carbonyl compounds with a protected amino group (*e.g*., a Boc-PG) as starting materials enables the synthesis of new derivatives. After removing the protecting group, these products can be used as building blocks in organic synthesis.

## 2 MATERIALS AND METHODS

### 2.1 Materials

Spectral analysis was provided by Enamine Ltd. (Ukraine). ^1^H and ^13^C NMR spectra were recorded on Bruker 170 Avance 500 (at 500 MHz for ^1^H and 126 MHz for ^13^C) and Bruker Avance 400 spectrometers (400 MHz for ^1^H and 100 MHz for ^13^C NMR) in DMSO-d_6_. The signals are given in the δ scale. Mass spectra were recorded on an Agilent 1100 High-Performance Liquid Chromatography (HPLC) equipped with a diode matrix and an Agilent LC/MSD SL mass-selective detector, a SUPELCO Ascentis Express C18 chromatographic column 2.7 μm 4.6 mm x 15 cm”. Control throughout the reaction and the individuality of the obtained substances was carried out by TLC method on silica gel-coated “Polychrome SI F254” plates with a fluorescent detector in the hexane-ethyl acetate 2:1 system, the developer was an ultraviolet lamp. If necessary, additional purification of the obtained compounds was carried out using flash chromatography (UPFP) on a PuriFlash XS520 Plus device using gradient elution. Elemental analysis was realized on a EuroVector EA-3000 instrument. The melting points of all synthesized compounds were determined using a Hanon Instruments MP450 open capillary tube automatic melting point apparatus.

All solvents and reagents were commercial grade and, if required, purified in accordance with the standard procedures. Precursors di-N-Boc-protected methyl-2-aminoacrylate **7** was synthesized as described elsewhere [15]. Starting a-aminoamidines **1a-c** were obtained by a known method [4]. Starting unsaturated ketones, **9a-e,** were prepared as described elsewhere [16].

### 2.2 Molecular Docking Setup

The preparation of the receptor and ligands was carried out with the AutoDock Tools (ADT) software, version 1.5.7 [17]. The addition of hydrogen and the calculation of the Gasteiger charges of the receptor and ligands were also performed using the ADT software. Molecular docking calculations were performed with the AutoDock Vina 1.1.2 software [18]. The 3D X-ray structure of phosphoinositide-3-kinase gamma (PI3Kγ) (PDB ID: 3SD5, 5JHA) [19, 20] and αβ-tubulin heterodimer (PDB ID: 5M7E) [20] were downloaded from the RCSB Protein Data Bank. Semi-flexible docking was performed so that the receptor was kept rigid and the ligand molecules were conformationally flexible. The size of the cubic box generated by the ADT software in the region of the receptor interaction was defined as 30×30×30 Å. For PI3K receptors, the center of the grid box at Cartesian coordinates was set to x= 22.36, y= 14.68, and z= 21.02 for PDB 3SD5, x= 24.02, y= -2.40 and z= 21.89 for PDB 5JHA with the grid point spacing set to 0.375 Å, respectively. For αβ-tubulin heterodimer, the chains A and B were used with the center of the grid box at Cartesian coordinates x= 17.37, y= 65.63, and z= 44.57, respectively. For all runs, the number of binding modes was set to 9 and the exhaustiveness to 256. For each ligand, three independent runs were performed using different random seeds. The best docking mode corresponds to the largest ligand-binding affinity. Molecular graphics and visualization were performed using VMD 1.9.3 [21].

## 3 EXPERIMENTAL

### 3.1 Synthesis of tert-butyl (2-(4-oxo-6-methyl-pyrimidin-2-yl)propan-2-yl)carbamate (3)

260 mg (1 mmol) of *tert*-butyl (1-amino-1-imino-2-methylpropan-2-yl)carbamate acetate **1a** and 130 mg (1 mmol) of ethyl acetoacetate **2** were dissolved in pyridine (5 mL), the mixture was heated at 75°C for 12 h. After completion of the reaction (TLC control), the solvent was removed under vacuum, brine (20 mL) was added, and compound **3** was extracted by ethyl acetate. The solvent was removed, and MTBE (20 mL) was added. The crude residue of **3** was filtered and washed with methanol (10 mL).

Yield: 182 mg (68%), beige solid, mp 143-144°C. ^1^H NMR (400 MHz, DMSO-*d_6_*) δ, ppm: 1.29 (s, 3H, CH_3_), 1.50 – 1.31 (m, 12H, COOC(CH_3_)_3_+CH_3_), 2.15 (s, 3H, CH_3_), 6.02 (s, 1H, CH), 6.9 (bs, 1H, NH), 11.9 (bs, 1H, NH). ^13^C NMR (126 MHz, DMSO-*d_6_*) δ, ppm: 23.5, 26.1, 28.0, 28.2, 54.9, 78.2, 110.1 (C^5^), 126.6, 154.1, 163.5, 164.7 (C^4^=O). Mass spectrum, m/z (Irel, %): 212.0 (17), 268.2 [M+H]^+^(100). Found, %: C 58.46; H 7.87; N 15.75. C_13_H_21_N_3_O_3_. Calculated, %: C 58.41; H 7.92; N 15.72.

N-(2-(4-oxo-5,6-dihydropyrimidin-2-yl)propan-2-yl)methanesulfonamide (**5**).

The **5** was synthesized by a procedure identical to the synthesis of compound **3** from 86 mg (1 mmol) of methyl acrylate **4**, 240 mg (1 mmol) of 2-methyl-2-(methylsulfonamido)propanimidamide acetate **1a,** and pyridine (5 mL). Yield 87 mg (37%), beige solid, mp 135-136°C. ^1^H NMR (400 MHz, DMSO-*d_6_*) δ, ppm: 1.43 (s, 6H, 2CH_3_), 2.23 (t, 2H, CH_2_), 2.94 (s, 3H, SO_2_CH_3_), 3.49 (t, 2H, CH_2_), 7.03 (bs, 1H, NH), 9.86 (bs, 1H, NH). ^13^C NMR (126 MHz, DMSO-*d_6_*) δ, ppm: 26.5, 29.3, 43.6, 43.9, 57.7, 157.9 (C^2^), 171.3 (C^4^=O). Mass spectrum, m/z (I_rel_, %): 234.0 [M+H]^+^(100). Found, %: C 41.17; H 6.42; N 18.05. C_8_H_15_N_3_O_3_S. Calculated, %: C 41.19; H 6.48; N 18.01.

Tert-Butyl (2-(4-oxo-5,6-dihydropyrimidin-2-yl)butan-2-yl)carbamate (**6**).

The **6** was synthesized by a procedure identical to the synthesis of compound **3** from 86 mg (1 mmol) of methyl acrylate **4**, 275 mg (1 mmol) of *tert*-butyl (1-amino-1-imino-2-methylbutan-2-yl)-carbamate acetate **1b** and pyridine (5 mL). Yield 220 mg (82%), white solid, mp 159-160°C. ^1^H NMR (400 MHz, DMSO-*d_6_*) δ, ppm: 0.65 (t, 3H, CH_3_), 1.37 (bs, 12H, COOC(CH_3_)_3_+CH_3_), 1.75 (q, 1H), 1.95 (q, 1H), 2.25 (t, 2H, CH_2_), 3.51 (t, 2H, CH_2_), 6.52 (bs, 1H, NH), 10.01 (bs, 1H, NH). ^13^C NMR (126 MHz, DMSO-*d_6_*) δ, ppm: 8.3, 22.7, 28.6, 29.4, 43.3 (C^6^), 57.8, 78.4, 153.9 (C=O), 157.5 (C^2^), 171.3 (C^4^=O). Mass spectrum, m/z (I_rel_, %): 270.2 [M+H]^+^(100). Found, %: C 58.02; H 8.60; N 15.63. C_13_H_23_N_3_O_3_. Calculated, %: C 57.97; H 8.61; N 15.60.

Tert-Butyl (tert-butoxycarbonyl)(2-(2-((tert-butoxycarbonyl)amino)propan-2-yl)-4-oxo-5,6-dihydropyrimidin-5-yl)carbamate (**8**).

The **8** was synthesized by a procedure identical to the synthesis of compound **3** from 300 mg (1 mmol) of N, N-di(*tert*-butoxycarbonyl)methyl 2-aminoacrylate **7**, 260 mg (1 mmol) of *tert*-butyl (1-amino-1-imino-2-methylpropan-2-yl)carbamate acetate **1a** and pyridine (5 mL). Yield 283 mg (60%), white solid, mp 162-163°C. ^1^H NMR (400 MHz, DMSO-*d_6_*) δ, ppm: 1.31 (s, 3H, CH_3_), 1.34 (s, 12H, COOC(CH_3_)_3_+CH_3_), 1.43 (s, 18H, 2(COOC(CH_3_)_3_)), 3.81 – 3.61 (m, 2H, CH_2_), 4.85 – 4.66 (m, 1H, CH), 6.63 (s, 1H, NH), 10.1 (bs, 1H, NH). ^13^C NMR (126 MHz, DMSO-*d_6_*) δ, ppm: 27.5, 27.8, 28.1, 47.0 (C^6^), 52.5, 54.4, 78.0, 82.6, 151.3, 153.9, 157.5(C^2^), 168.2 (C^4^=O). Mass spectrum, m/z (Irel, %): 471.2 [M+H]^+^(100). Found, %: C 56.11; H 8.18; N 11.90. C_22_H_38_N_4_O_7_. Calculated, %: C 56.15; H 8.14; N 11.91.

### 3.2 Synthesis of N-(2-(4,6-diphenylpyrimidin-2-yl)propan-2-yl)methanesulfonamide (10a)

240 mg (1 mmol) of 2-methyl-2-(methylsulfonamido)-propanimidamide acetate **1a** and 210 mg (1 mmol) of 1,3-diphenylprop-2-en-1-one (chalcone) **9a** were dissolved in pyridine (10 mL), the mixture is stirred at 100°C for 24 hours. The solvent was removed under vacuum, and 10 mL of methanol was added to the dry residue. The formed precipitate was filtered and washed with methanol. Yield 310 mg (81%), beige solid, mp 164-166°C. ^1^H NMR (400 MHz, DMSO-d_6_) δ, ppm: 1.79 (s, 6H, 2CH_3_), 2.83(s, 3H, SO_2_CH_3_), 7.64-7.53 (m, 7H, Ar^+^NH), 8.45-8.38 (m, 4H, Ar), 8.47 (s, 1H, CH). ^13^C NMR (126 MHz, DMSO-d_6_) δ, ppm: 29.0, 44.2, 60.9, 110.5 (C^5^), 127.9, 129.4, 131.6, 137.1, 164.2 (C^4^+C^6^), 173.0 (C^2^). Mass spectrum, m/z (Irel, %): 368.0 [M+H]^+^(100). Found, %: C 65.35; H 5.74; N 11.44. C_20_H_21_N_3_O_2_S. Calculated, %: C 65.37; H 5.76; N 11.44.

Substances **10b–i** were synthesized from corresponding 1,3-diarylprop-2-en-1-one **9b-e** using a method identical to the synthesis of substance **10a**.

N-(2-(4-(4-chlorophenyl)-6-phenylpyrimidin-2-yl)propan-2-yl)-methanesulfon-amide (**10b**).

Yield: 350 mg (87%), beige solid, mp 186-187°C. ^1^H NMR (400 MHz, DMSO-*d*_6_) δ, ppm: 1.78 (s, 6H, 2CH_3_), 2.82 (s, 3H, SO_2_CH_3_), 7.62-7.56 (m, 4H, Ar+NH), 7.65 (d, *J* = 8.5 Hz, 2H, Ar), 8.45-8.39 (m, 2H, Ar), 8.47 (d, *J* = 8.5 Hz, 2H, Ar), 8.49 (s, 1H, CH). ^13^C NMR (126 MHz, DMSO-*d_6_*) δ, ppm: 29.0, 44.3, 60.9, 110.5 (C^5^), 127.9, 129.4, 129.8, 131.7, 135.9, 136.5, 136.9, 162.9 (C^4^), 164.4 (C^6^), 173.1 (C^2^). Mass spectrum, m/z (Irel, %): 404.0 (20), 402.0 [M+H]^+^(100). Found, %: C 59.75; H 5.00; N 10.51. C_20_H_20_ClN_3_O_2_S. Calculated, %: C 59.77; H 5.02; N 10.46.

N-(2-(4-(3-bromophenyl)-6-(4-bromophenyl)pyrimidin-2-yl)propan-2-yl)-methanesulfon-amide (**10c**).

Yield: 390 mg (74%), beige solid, mp 230-231°C. ^1^H NMR (400 MHz, DMSO-*d*_6_) δ, ppm: 1.77 (s, 6H, 2CH_3_), 2.81 (s, 3H, SO_2_CH_3_), 7.55 (t, 1H, Ar), 7.67 (s, 1H, Ar), 7.78 (t, *J* = 7.8 Hz, 3H, Ar), 8.41 (d, *J* = 8.3 Hz, 2H, Ar), 8.45 (d, *J* = 7.8 Hz 1H, Ar), 8.55 (s, 1H, NH), 8.66 (s, 1H, CH). ^13^C NMR (126 MHz, DMSO-*d_6_*) δ, ppm: 28.9, 44.3, 60.8, 110.8 (C^5^), 123.0, 125.6, 126.9, 130.0, 130.5, 131.5, 132.4, 134.3, 136.1, 139.3, 162.8 (C^4^), 163.3 (C^6^), 173.3 (C^2^). Mass spectrum, m/z (Irel, %): 524.0 [M+H]^+^(100), 526.0 (50). Found, %: C 45.75; H 3.66; N 7.95. C_20_H_19_Br_2_N_3_O_2_S. Calculated, %: C 45.73; H 3.65; N 8.00.

N-(2-(4,6-bis(4-methoxyphenyl)pyrimidin-yl)propan-2-yl)methanesulfonamide (**10d**).

Yield: 188 mg (44%), light yellow solid, mp 132-133°C. ^1^H NMR (400 MHz, DMSO-*d*_6_) δ, ppm: 1.76 (s, 6H, 2CH_3_), 2.81 (s, 3H, SO_2_CH_3_), 3.86 (s, 6H, 2OCH_3_), 7.12 (d, *J* = 8.5 Hz, 4H, Ar), 7.51 (s, 1H, NH), 8.31 (s, 1H, CH), 8.40 (d, *J* = 8.4 Hz, 4H, Ar). ^13^C NMR (126 MHz, DMSO-*d_6_*) δ, ppm: 29.0, 44.1, 55.9, 60.9, 108.6 (C^5^), 114.7, 129.5, 129.6, 162.2 (C^4^), 163.5 (C^6^), 172.5 (C^2^). Mass spectrum, m/z (Irel, %): 428.2 [M+H]^+^(100), 430.2 (20). Found, %: C 61.80; H 5.92; N 9.84. C_22_H_25_N_3_O_4_S. Calculated, %: C 61.81; H 5.89; N 9.83.

N-(2-(4-(4-Chlorophenyl)-6-(4-methoxyphenyl)-pyrimidin-2-yl)propan-2-yl)methanesulfon-amide (**10e**).

Yield: 380 mg (88%), beige solid, mp 202-204°C. ^1^H NMR (400 MHz, DMSO-*d*_6_) δ, ppm: 1.77 (s, 6H, 2CH_3_), 2.82 (s, 3H, SO_2_CH_3_), 3.86 (s, 3H, OCH_3_), 7.12 (d, *J* = 8.5 Hz, 2H, Ar), 7.57 (s, 1H, NH), 7.64 (d, *J* = 8.2 Hz, 2H, Ar), 8.45-8.36 (m, 4H, Ar), 8.46 (s, 1H, CH). ^13^C NMR (126 MHz, DMSO-*d_6_*) δ, ppm: 28.9, 44.2, 55.9, 60.9, 109.6 (C^5^), 114.7, 129.2, 129.4, 129.7, 136.0, 136.4, 162.4, 162.6 (C^4^), 164.0 (C^6^), 172.9 (C^2^). Mass spectrum, m/z (Irel, %): 432.2 [M+H]^+^(100), 434.0 (40). Found, %: C 58.43; H 5.10; N 9.71. C_21_H_22_ClN_3_O_3_S. Calculated, %: C 58.40; H 5.13; N 9.73.

Tert-Butyl (2-(4,6-diphenylpyrimidin-2-yl)butan-2-yl)-carbamate (**10f**).

Yield: 200 mg (49%), beige solid, mp 124-125°C. ^1^H NMR (500 MHz, DMSO-*d*_6_) δ, ppm: 0.74 (t, 3H, CH_3_), 1.36 (bs, 9H, COOC(CH_3_)_3_), 1.68 (s, 3H, CH_3_), 2.20-2.07 (m, 2H, CH_2_), 7.06 (bs, 1H, NH), 7.64-7.54 (m, 6H, Ar), 8.38-8.34 (m, 4H, Ar), 8.41 (s, 1H, CH). ^13^C NMR (126 MHz, DMSO-*d*_6_) δ, ppm: 8.8, 25.2, 32.2, 60.8, 77.9, 110.2, 127.8, 129.3, 131.5, 137.2 (C=O), 154.9 (C^2^), 164.0 (C^4^+C^6^). Mass spectrum, m/z (Irel, %): 349.2(20), 404.2 [M+H]^+^(100), 405.2(20), 406.2(5). Found, %: C 74.42; H 7.21; N 10.38. C_25_H_29_N_3_O_2_. Calculated, %: C 74.41; H 7.24; N 10.41.

Tert-Butyl (2-(4-(4-chlorophenyl)-6-phenylpyrimidin-2-yl)butan-2-yl)carbamate (**10g**).

Yield: 360 mg (82%), beige solid, mp 126-127°C. ^1^H NMR (500 MHz, DMSO-d6) δ, ppm: 0.75 (t, 3H, CH_3_), 1.35 (bs, 9H, COOC(CH_3_)_3_), 1.67 (s, 3H, CH_3_), 2.18-2.05 (m, 2H, CH_2_), 7.06 (bs, 1H, NH), 7.62-7.53 (m, 3H, Ar), 7.65 (d, *J* = 8.7 Hz, 2H, Ar), 8.37 (d, 2H, Ar), 8.40 (d, *J* = 8.7 Hz, 2H, Ar), 8.45 (s, 1H, CH). ^13^C NMR (126 MHz, DMSO-*d*_6_) δ, ppm: 8.8, 25.1, 28.7, 60.8, 77.9, 110.2, 127.8, 129.3, 129.4, 129.6, 131.6, 136.1, 136.4, 137.1 (C=O), 155.0 (C^2^), 162.8 (C^6^), 164.2 (C^4^). Mass spectrum, m/z (Irel, %): 384.5(10), 438.2 [M+H]^+^(100), 440.2(40), 441.2(15). Found, %: C 68.58; H 6.41; N 9.60. C_25_H_28_ClN_3_O_2_. Calculated, %: C 68.56; H 6.44; N 9.59.

Tert-Butyl-(2-(4-(3-bromophenyl)-6-(4-bromophenyl)pyrimidin-2-yl)butan-2-yl)carbamate (**10h**).

Yield: 360 mg (64%), beige solid, mp 165-166°C. ^1^H NMR (500 MHz, DMSO-*d*_6_) δ, ppm: 0.78 (t, 3H, CH_3_), 1.35 (bs, 9H,COOC(CH_3_)_3_), 1.65 (s, 3H, CH_3_), 2.18-2.05 (m, 2H, CH_2_), 7.12 (bs, 1H, NH), 7.54 (t, *J* = 7.9 Hz, 1H, Ar), 7.78 (dd, *J* = 10.8 Hz, *J* = 8.7 Hz, 3H, Ar), 8.35 (d, *J* = 8.7 Hz, 2H, Ar), 8.39 (d, *J* = 6.8 Hz, 1H, Ar), 8.51 (s, 1H, Ar), 8.58 (s, 1H, CH). ^13^C NMR (126 MHz, DMSO-*d_6_*) δ, ppm: 8.7, 24.9, 28.7, 60.8, 77.9, 110.4, 122.9, 125.4, 126.9, 129.9, 130.3, 131.5, 132.4, 134.2, 136.3, 139.5 (C=O), 155.1 (C^2^), 162.6 (C^6^), 163.2 (C^4^). Mass spectrum, m/z (Irel, %): 562.0[M+H]^+^(100), 564.0(50), 561.2(50). Found, %: C 53.45; H 4.85; N 7.50. C_25_H_27_Br_2_N_3_O_2_. Calculated, %: C 53.49; H 4.85; N 7.49.

Tert-Butyl (2-(4-(4-chlorophenyl)-6-(4-methoxyphenyl)-pyrimidin-2-yl)butan-2-yl)carbamate (**10i**).

Yield: 100 mg (21%), beige solid, mp 139-140°C. ^1^H NMR (500 MHz, DMSO-*d*_6_) δ, ppm: 0.73 (t, 3H, CH_3_), 1,36 (bs, 9H, COOC(CH_3_)_3_), 1.66 (s, 3H, CH_3_), 2.09 (m, 2H, CH_2_), 3.86 (s, 3H, OCH_3_), 7.03 (bs, 1H, NH), 7.12 (d, *J* = 8.7 Hz, 2H, Ar), 7,64 (d, *J* = 8.7 Hz, 2H, Ar), 8.35 (d, *J* = 8.7 Hz, 2H, Ar), 8.37 (s, 1H, CH), 8.38 (d, *J* = 8.7 Hz, 2H, Ar). ^13^C NMR (126 MHz, DMSO-*d_6_*) δ, ppm: 8.8, 25.2, 28.7, 55.9, 60.8, 77.9, 109.3, 114.7, 129.4, 129.5, 136.2 (C=O), 154.9 (C^2^), 162.3, 162.5 (C^6^), 163.8 (C^4^). Mass spectrum, m/z (Irel, %): 468.2[M+H]^+^(100), 470.2(40). Found, %: C 66.70; H 6.42; N 9.03. C_26_H_30_ClN_3_O_3_. Calculated, %: C 66.73; H 6.46; N 8.98.

### 3.3 Synthesis of 2-(4,6-diphenylpyrimidin-2-yl)butan-2-amine hydrochloride (10k)

40 mg (0.1 mmol) of *tert*-butyl (2-(4,6-diphenylpyrimidin-2-yl)butan-2-yl)carbamate **10f** was dissolved in methanol (5 mL) and concentrated hydrochloric acid (0.2 mL) was added. The reaction mixture was stirred at 40°C for 6 h, the solvent was removed under vacuum, and the solid was washed dry acetonitrile. Yield: 28 mg (83%), white solid, mp 136-137°C. ^1^H NMR (400 MHz, DMSO-*d_6_*) δ, ppm: 0.81 (t, 3H, CH_3_), 1.78 (s, 3H, CH_3_), 2.22-2.10 (m, 2H, CH_2_), 7.61-7.56 (m, 6H, Ar), 8.51-8.49 (m, 4H, Ar), 8.59 (s, 1H, CH), 8.84 (bs, 3H, NH_2_^.^HCl). ^13^C NMR (126 MHz, DMSO-*d_6_*) δ, ppm: 7.9, 23.7, 32.3, 61.0, 110.8 (C^5^), 127.7, 128.9, 131.5, 135.9, 164.2 (C^4^+C^6^), 168.1 (C^2^). Mass spectrum, m/z (Irel, %): 340.0 [M+H]^+^(100). Found, %: C 70.65; H 6.50; N 12.39. C_20_H_22_ClN_3_. Calculated, %: C 70.68; H 6.52; N 12.36.

Substances 11 and 12 were synthesized using a procedure identical to the synthesis of compound **10k**.

2-(2-Aminopropan-2-yl)-6-methylpyrimidin-4(3H)-one dihydrochloride (**11**).

Yield: 19 mg (78%), white solid, mp 145°C. ^1^H NMR (500 MHz, DMSO-*d_6_*) δ, ppm: 1.60 (s, 6H, 2CH_3_), 2.26 (s, 3H, CH_3_), 6.33 (s, 1H, CH), 7.52 (bs, 3H, NH_3_Cl), 8.7 (bs, 2H, NH_2_Cl). ^13^C NMR (126 MHz, DMSO-*d_6_*) δ, ppm: 22.8, 24.7, 56.0, 108.9 (C^5^), 161.0 (C^2^), 163.6 (C^6^), 165.6 (C^4^=O), Mass spectrum, m/z (Irel, %): 168.2 [M+H]^+^(100), 169.2(10). Found, %: C 40.05; H 6.27; N 17.55. C_8_H_15_Cl_2_N_3_O. Calculated, %: C 40.02; H 6.30; N 17.50.

Methyl 3-((1,2-diamino-2-methylbutylidene)amino)-propanoate dihydrochloride (**12**).

Yield: 15 mg (71%), white solid, mp 51-52°C. ^1^H NMR (500 MHz, DMSO-*d_6_*) δ, ppm: 0.82 (t, 3H, CH_3_), 1.62 (s, 3H, CH_3_), 1.97 (q, 1H, CH_2_), 2.11 (q, 1H, CH_2_), 2.78-2.73 (m, 2H, CH_2_), 3.58-3.51 (m, 2H, CH_2_), 3.60 (s, 3H, OCH_3_), 9.76-9.35 (m, 4H, 2NH_2_^.^HCl). ^13^C NMR (126 MHz, DMSO-*d_6_*) δ, ppm: 7.5, 20.6, 30.8, 31.2, 38.4, 51.6 (OCH_3_), 59.3, 165.2 (C=N), 170.8 (C=O). Mass spectrum, m/z (Irel, %): 202.2 [M+H]^+^(100). Found, %: C 39.40; H 7.75; N 15.31. C_9_H_21_Cl_2_N_3_O_2_. Calculated, %: C 39.43; H 7.72; N 15.33 (**Supplementary information**).

## 4 RESULTS AND DISCUSSION

**Scheme 1:**
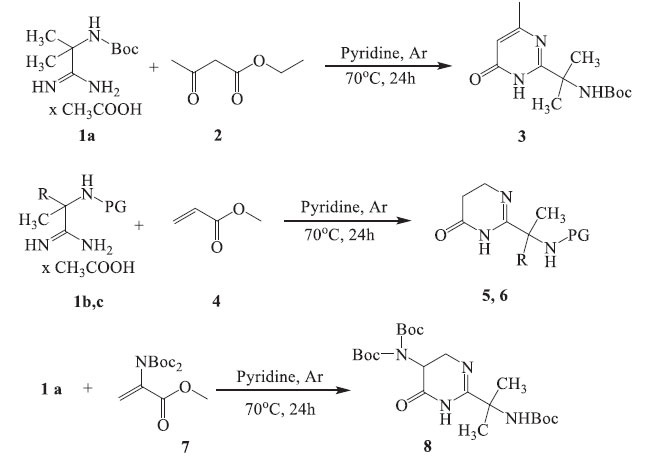
Synthesis of pyrimidin-4(3H)-one **3** and 5,6-dihydropyrimidin-4(3H)-one derivatives **5-6, 8**.

We carried out a study on the reaction between α-aminoamidines and other unsaturated carbonyl compounds. Our results showed that when Boc-protected α-aminoamidine **1a** reacted with ethyl acetoacetate **2,** the treatment resulted in the formation of *tert-*butyl (2-(4-oxo-6-methyl-pyrimidin-2-yl)propan-2-yl)carbamate **3**, as summarized in Scheme **1**. This reaction proceeded under the conditions used in our previous studies [4, 6] and involved the heating of a stoichiometric mixture of the starting compounds in pyridine at 70°C in an inert atmosphere (under Ar) for 24 hours. When the α-aminoamidines **1b** and **1c** reacted with methyl acrylate **4**, dihydro derivatives of 4(3H)pyrimidinone were formed. These derivatives were identified as N-(2-(4-oxo-5,6-dihydropyrimidin-2-yl)propan-2-yl)methanesulfonamide **5** and *tert-*butyl (2-(4-oxo-5,6-dihydropyrimidin-2-yl)butan-2-yl)carbamate **6**, respectively. Similarly, when α-aminoamidine **1a** was reacted with the two-*Boc* derivative of aminoacrylic acid **7**, the resulting product was identified as *tert-*Butyl (*tert*-butoxycarbonyl)(2-(2-((tert-butoxycarbonyl)amino)propan-2-yl)-4-oxo-5,6-dihydropyrimidin-5-yl)carbamate **8**.

**Scheme 2:**
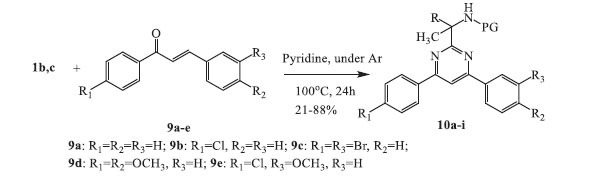
Synthesis of 2-(4,6-diphenylpyrimidin-2-yl)propan-2-amine derivatives **10a-i**.

We also studied the possibility of obtaining 2-methylamino-4,6-diaryl-substituted pyrimidines by cyclocondensation of α-aminoamidines **1** with various aryl-substituted unsaturated ketones (Scheme **2**). It was found that the reaction of acetates of α-aminoamidines **1b,c** with chalcones **9a-e** resulted in the production of derivatives of 2-(4,6-diphenylpyrimidin-2-yl)propan-2-amine **10a-i**. The treatment was carried out by heating a mixture of the starting compounds in a stoichiometric ratio in pyridine at 100°C in an inert atmosphere (under Ar) for 24 hours. Most of the target compounds were obtained in satisfactory to good yields (21-88%) (Table **1**).

**Scheme 3:**
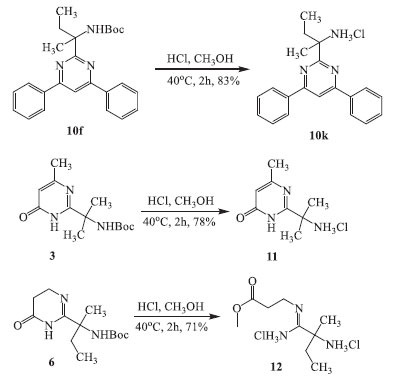
Removing the protecting groups in **3, 6,** and **10f**.

Compounds **3** and **10f** were subjected to deprotection using known protocols [4] to form unprotected pyrimidinone **11** and pyrimidine **10k** (Scheme **3**). All attempts to deprotect the Boc-amino dihydropyrimidines **6** and **8** under similar conditions were unsuccessful. The use of hydrochloric, trifluoroacetic, and sulfuric acids at room temperature and 40°C to deprotect compound **6** also did not lead to a deprotected amine but to a mixture of unidentified compounds. In the case of Boc-protected compound **8**, heating in methanol in the presence of hydrochloric acid at 40 °C finally gave the product of opening the pyrimidinone ring in the form of ester **12**.

The pectral methods of 1H proved the structure of all obtained target compounds- and ^13^C-NMR spectroscopy and mass spectrometry. The signals of the molecular ions of the compounds or the amine hydrochlorides characterize the mass spectra of the obtained target compounds. The ^1^H NMR spectrum of compound **3** is characterized by the proton signal of the pyrimidine ring at 6.02 ppm, the signal of the amino group at 6.90 ppm, and the signals for terminal substituents and the protecting Boc-group. The ^1^H NMR spectra of compounds **5-6** are characterized by signals of CH_2_-CH_2_ protons at 1.75-2.23 and 3.49-3.51 ppm and other corresponding proton signals. The ^1^H NMR spectra of compound **8** show signals of CH_2_-CH protons at 3.64-3.78, 4.76 ppm, and corresponding protons of substituents and protecting Boc-groups. The ^1^H NMR spectra of compounds **10a-i** are characterized by proton signals of the pyrimidine ring at 8.66 - 8.40 ppm, signals of the amino group, and the signals of the corresponding aromatic and aliphatic protons. The structure of compound **12** was established on the basis of ^1^H- and ^13^C-NMR spectra containing signals from protons of the methoxy group at 3.60 ppm, signals from CH_2_-CH_2_ protons at 3.53-3.57 and 1.97-2.11 ppm and other corresponding proton signals, carbon atom signal from the methoxy group at 51.6 ppm and other corresponding signals, and mass spectrometry data characterized by a molecular ion signal at 202.2 [M+H]^+^.

**Scheme 4:**
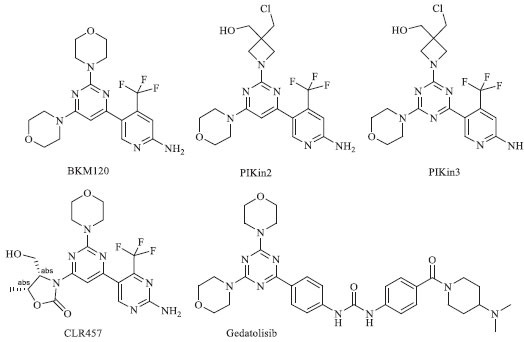
Chemical structures of known PI3K inhibitors.

**Molecular docking calculations:** Molecular docking calculations have become an essential complementary tool to outline the biological activity of given compounds [22]. However, docking calculations rely strongly on comparison with available experimental data and standard inhibitors for a certain receptor [23-26]. The 2-amino pyrimidines and their 4,6-disubstituted analogs have been found to strongly inhibit the phosphoinositide-3-kinase (PI3K) family of lipid kinases (Scheme **4**) [19, 27-37]. The activity of PI3K enzymes is crucial in cancer development, with the PI3Kγ isoform being a particularly promising target for the treatment of various types of cancer, including ovarian, breast, prostate, stomach, colorectal, glioblastoma, endometrial, and brain cancer [31, 33, 38-42]. Buparlisib (BKM120) is a highly advanced orally bioavailable pan-PI3K inhibitor currently being evaluated in several clinical trials [19, 27, 32, 33, 37, 43]. Recent *in vitro* and in-cell studies, along with X-ray analysis, have elucidated Buparlisib's mechanism of action against PI3K and tubulin [20]. Although Buparlisib is an advanced PI3K inhibitor, it has an off-target effect on microtubule polymerization [44]. As a result, chemical derivatives PIKin2 and PIKin3 have been developed (Scheme **4**) to specifically target PI3K and tubulin inhibitions separately [20, 32].

The binding mode of BKM120 and PIKin2 inhibitors has been determined in their cocrystals with the PIK3γ receptor, as illustrated in Supplemental Figure **S1A-B**. Both ligands bind to the well-defined binding site, which can aid in setting up molecular docking calculations.

**Fig. (1):**
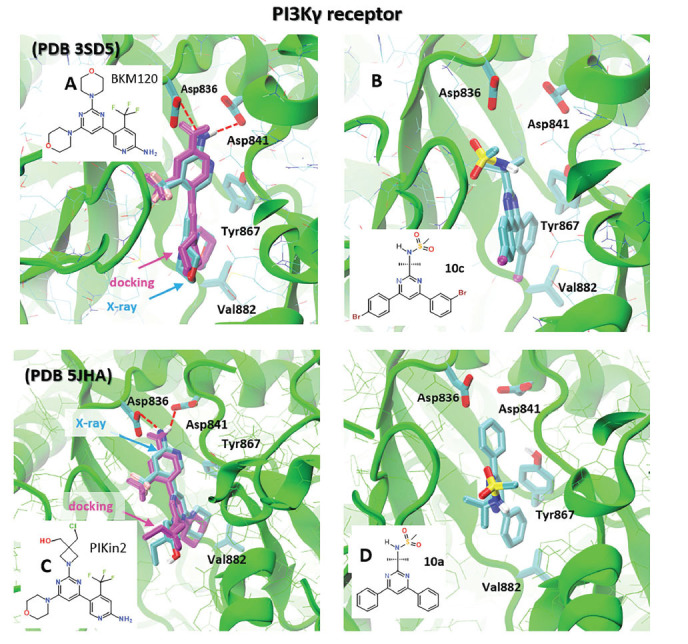
Comparison between the best docking mode of BKM120 and PIKin2 (magenta) and its X-ray structure (cyan) in phosphoinositide 3-kinase gamma (PI3Kγ) receptor from two different X-ray structures (**A**) PDB 3SD5 and (**C**) PDB 5JHA, respectively. The docking configuration of the best-binding ligands **10c** (**B**, PDB 3SD5) and **10a** (**D**) PDB 5JHA) in the active center of the PI3Kγ receptor.

The inhibitory strength, binding affinity, and selectivity of the newly synthesized derivatives **10a-k** towards the PI3Kγ enzyme were evaluated using molecular docking calculations. Unprotected pyrimidinone **11** was also taken into account. Additionally, the calculated binding affinity was compared to that of some existing inhibitors (Scheme **4**) with known experimental anticancer activity, as summarized in Table **2**. The two crystallographic structures of the PI3Kγ receptor (PDB IDs: 3SD5 and 5JHA) were considered for further molecular docking calculations (Fig. **1**).

The first step involved re-docking well-known inhibitors, BKM120 and PIKin2, against the corresponding PIK3γ receptors to establish a baseline for our docking process and parameters. This procedure is crucial because it validates the used docking scoring function and parameters [23, 24, 26]. Fig. (**1A** and **C**) illustrate the comparison between the position of BKM120 and PIKin2 in its co-crystal with PIK3γ and their best binding mode as determined by molecular docking calculations. Both X-ray and docked structures exhibit significant overlap (Fig. **1A**). BKM120 binds to the receptor with one of the morpholine rings to the hinge at receptor residue Val882, and the terminal aminopyridine group of the ligand forms hydrogen bonds with the receptor residues Asp836, Asp841 and Tyr867 (Fig. **1A**) [27]. A similar binding mode was observed for inhibitor PIKin2, as illustrated in Fig. (**1C**).

Next, molecular docking calculations were performed for our new derivatives **10a-k** against PIK3γ. Table **2** demonstrates that the binding affinity of ligands **10a-k** towards PIK3γ (PDB 3SD5) varies in a range from -8.5 kcal/mol up to -9.1 kcal/mol. An analysis of the short contacts and intermolecular interactions of the hit ligand **10c** with the nearest PI3K amino acid residues (Fig. **1B**) showed that its binding mode overlaps and occupies the binding space of the original inhibitor BKM120. Ligand **10c** displayed hydrophobic interactions with Trp867 and Val882, which are the main driving force for the strong binding. Furthermore, **10c** exhibited strong interactions with Asp836 and Asp841 through ionic strength (Fig. **1D**). It should be noted that pyrimidine-4 (3H)-one derivative **11** and unprotected 5,6-dihydropyridine derivative **8**, which lack the scaffold of BKM120 and PIKin2, revealed a rather weak inhibitory potency (Table **2**).

The binding affinity of ligands **10a-k** towards PIK3γ, estimated using another X-ray receptor structure (PDB 5JHA), revealed the larger variation being in a range from -7.7 kcal/mol up to -9.2 kcal/mol, as seen in Table **2**. For this X-ray receptor structure, the best binding ligand is **10a**, which displayed a similar binding mode to that of PIKin2 (Fig. **1D**). Ligand **10a** is also characterized by hydrophobic interactions with Trp867 and Val882. Polar residues Asp836 and Asp841 also play an essential role in stabilizing ligand **10a** in the active site of PIK3γ.

In terms of the binding affinity towards PIK3γ, the majority of the synthesized derivatives **10a-k** demonstrated the binding energies exciding the threshold of -8.5 kcal/mol (Table **2**), which is an excellent finding compared to the binding affinity of the available inhibitors, such as PIKin2 and PIKin3 (Scheme **4**). The newly synthesized derivatives **10a**, **10b,** and **10c** are characterized with the same affinity level as the well-known inhibitors, such as CLR457 and Gedatolisib (Scheme **4** and Table **2**).

Buparlisib has become the golden standard for inhibiting PI3K and treating cancer [20, 32, 43]. However, there are some debates about its off-target effect, indicating that the antiproliferative activity of BKM120 might mainly be due to microtubule-dependent cytotoxicity rather than through inhibition of PI3K. Therefore, developing next-generation PI3K inhibitors focuses on the dual activity with discrete PI3K and tubulin inhibition [20, 43].

With this context, we analyzed the inhibitory potency of the newly synthesized derivatives against the αβ-tubulin receptor, too. Recently, the X-ray structure of the BKM120–tubulin complex has become available [20]. The cocrystallized complex provides valuable information about ligand-receptor interactions and demonstrates that the orientation of the BKM120 ligand within its binding site was governed by hydrophobic interactions with neighbor receptor residues (Supplemental Fig. **S2**).

**Fig. (2):**
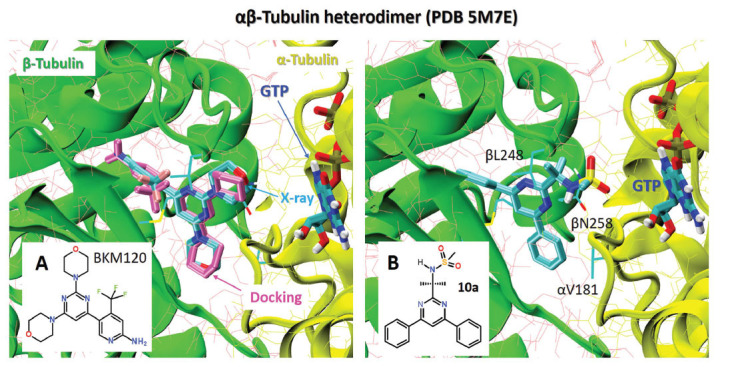
(**A**) Comparison of the best docking mode of BKM120 (magenta) with its X-ray structure (cyan) in αβ-tubulin heterodimer (PDB 5M7E). (**B**) The best docking mode of **10a** in the active center of the tubulin receptor.

The re-docking BKM120 and other available PIK3γ inhibitors (Scheme **4**) against the αβ-tubulin heterodimer demonstrated that they all revealed a high binding affinity of about -10 kcal/mol (Fig. **2** and Table **3**). The comparison between the binding position of BKM120 in its cocrystal with αβ-tubulin and its best binding docking mode confirmed that it occupies a GTP-binding pocket with a good overlap, as seen in Fig. (**2A**).

The molecular docking calculations of the ligands **10a-k** and some synthetic intermediates **8**, **11** demonstrated that most of the 4,6-diphenyl pyrimidine derivatives **10** are characterized with the inhibitory potency exciding those of the well-known BKM120 and PIKin3 (Table **3**). The best binding ligands, **10a** and **10b,** have the binding affinity of -10.7 and 10.4 kcal/mol (Fig. **2B**) so they can be promising leads for the development of pyrimidine-based potent microtubule-targeting agents [45].

## CONCLUSION

4,6-Diaryl-substituted pyrimidines have shown high inhibitory potency against phosphoinositide-3-kinases (PI3Ks), which are important oncology targets. Inhibiting PI3Ks could potentially offer a viable therapy for human cancers. In this study, we discuss the synthesis of a series of 2,4-diaryl-substituted pyrimidines and pyrimidinone derivatives. These compounds were obtained by cyclocondensation of α-aminoamidines with various saturated carbonyl derivatives and their analogs. The ability to vary the substituents in the obtained compounds suggests the potential to modulate their therapeutic properties by adjusting the bulky substituents in the 2-position. Molecular docking studies suggest that the synthesized derivatives exhibit high binding affinity toward PIK3γ, indicating their potential as promising candidates for the development of new anticancer agents.

## Figures and Tables

**Table 1:** Characteristics of the obtained compounds 3, 5-6, 8, 11-12, and 10a-k.

**Substance 3, 5-11**	**Protecting Group (PG)**	**R**	**R1**	**R2**	**R3**	**Yield, %**	**MP, °C**
3	Boc	CH_3_	-	-	-	68	143-144
5	Mz	CH_3_	-	-	-	37	135-136
6	Boc	CH_2_CH_3_	-	-	-	82	159-160
8	Boc	CH_3_	-	-	-	60	162-163
10a	Mz	CH_3_	H	H	H	81	164-166
10b	Mz	CH_3_	Cl	H	H	87	186-187
10c	Mz	CH_3_	Br	H	Br	74	230-231
10d	Mz	CH_3_	OCH_3_	OCH_3_	H	44	132-133
10e	Mz	CH_3_	Cl	OCH_3_	H	88	202-204
10f	Boc	CH_2_CH_3_	H	H	H	49	124-125
10g	Boc	CH_2_CH_3_	Cl	H	H	82	126-127
10h	Boc	CH_2_CH_3_	Br	H	Br	64	165-166
10i	Boc	CH_2_CH_3_	Cl	OCH_3_	H	21	139-140
10k	-	CH_2_CH_3_	H	H	H	83	136-137
11	-	CH_3_	-	-	-	78	145
12	-	CH_2_CH_3_	-	-	-	71	51-52

**Table 2:** The binding affinity of studied ligands with phosphoinositide 3-kinases gamma estimated by molecular docking calculations.

**Ligand**	**Binding Affinity to Different PI3Kγ Receptors (kcal/mol)**	
	PDB 3SD5	PDB 5JHA
BKM120	-9.4	-9.6
PIKin2	-8.3	-8.7
PIKin3	-8.0	-8.5
CLR457	-8.9	-9.0
Gedatolisib	-8.5	-8.8
**8**	-7.3	-7.2
**10a**	-8.6	-9.2
**10b**	-8.7	-8.7
**10c**	-9.1	-8.7
**10d**	-8.5	-7.7
**10e**	-8.6	-7.9
**10f**	-8.6	-8.8
**10g**	-8.7	-8.5
**10h**	-8.3	-8.7
**10i**	-8.6	-8.1
**10k**	-8.3	-8.5
**11**	-5.5	-5.6

**Table 3:** The binding affinity of studied ligands towards αβ-tubulin heterodimer (PDB 5M7E) was estimated by molecular docking calculations.

**Ligand**	**Binding Affinity (kcal/mol)**
BKM120	-10.2
PIKin2	-9.9
PIKin3	-10.1
CLR457	-10.1
**8**	-8.4
**10a**	-10.7
**10b**	-10.4
**10c**	-9.0
**10d**	*
**10e**	-9.0
**10f**	-9.8
**10g**	-9.6
**10h**	-9.1
**10i**	-8.7
**10k**	-10.1
**11**	-5.9

**Note:** *Ligand 10d binds outside the receptor pocket.

## Data Availability

The authors confirm that the data supporting the findings of this study are available within the article and it’s supplementary material.

## LIST OF ABBREVIATIONS

DMSO: Dimethyl Sulfoxide
MTBE: Methyl Tert-Butyl Ether
TLC: Thin Layer Chromatography
