# Supplementary material for: Synthesis of Novel Derivatives of 4,6-Diarylpyrimidines and Dihydro-Pyrimidin-4-one and In Silico Screening of Their Anticancer Activity
Source: Curr Org Synth. 2024 Dec 5;22(4):556–67. doi: 10.2174/0115701794356958241024082646 (PMC12272067; doi:10.2174/0115701794356958241024082646)
Supplement: Supplementary file 1 [file COS-22-4-556_SD1.pdf]

Supplementary Material

Synthesis of Novel Derivatives of 4,6-Diarylpyrimidines and Dihydro-Pyrimidin-4-one and *In Silico* Screening of Their Anticancer Activity

Oleksandr V. Onipko[3], Veronika Stoianova[1], Oleksandr V. Buravov[2,3], Valentyn A. Chebanov[1,2], Alexander Kyrychenko[1,2] and Eugene S. Gladkov[1,2,*]

[1]Institute of Chemistry and School of Chemistry, V. N. Karazin Kharkiv National University, 4 Svobody Sq., Kharkiv 61022, Ukraine; [2]State Scientific Institution "Institute for Single Crystals", Institute of Functional Materials Chemistry of National Academy of Sciences of Ukraine, 60 Nauky Ave., Kharkiv 61072, Ukraine; [3]Enamine Ltd., 67 Winston Churchill St., Kyiv 02660, Ukraine

CONTENTS:

Supplemental Figures S1 and S2 **2**

^{1}H and ^{13}C NMR spectra of **3, 5, 6, 8, 10a-k, 11** and **12** **3**

Mass spectra of **3, 5, 6, 8, 10a-k, 11** and **12** **19**

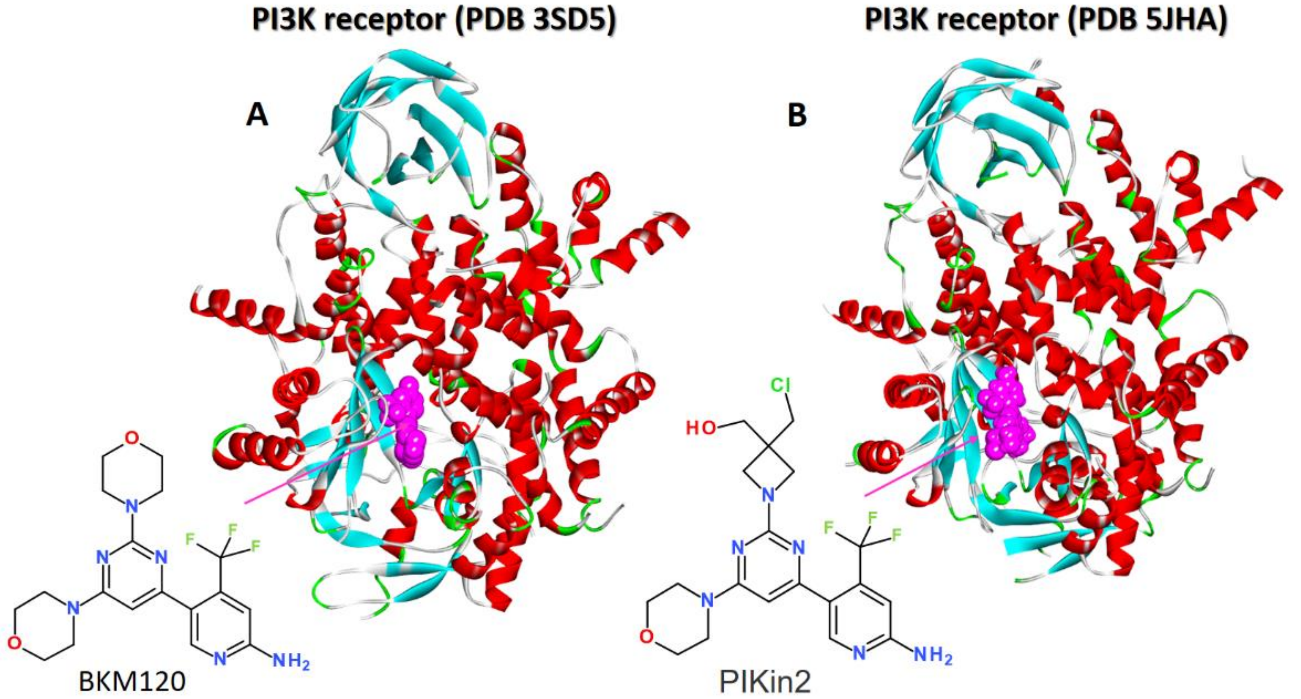

Fig. (S1). X-ray structure of phosphoinositide 3-kinase gamma (PI3Kγ) in complex with the potent inhibitor BKM120 (Buparlisib) (PDB 3SD5) (A) [1] an PIKin2 (PDB 5JHA) (B) [2].

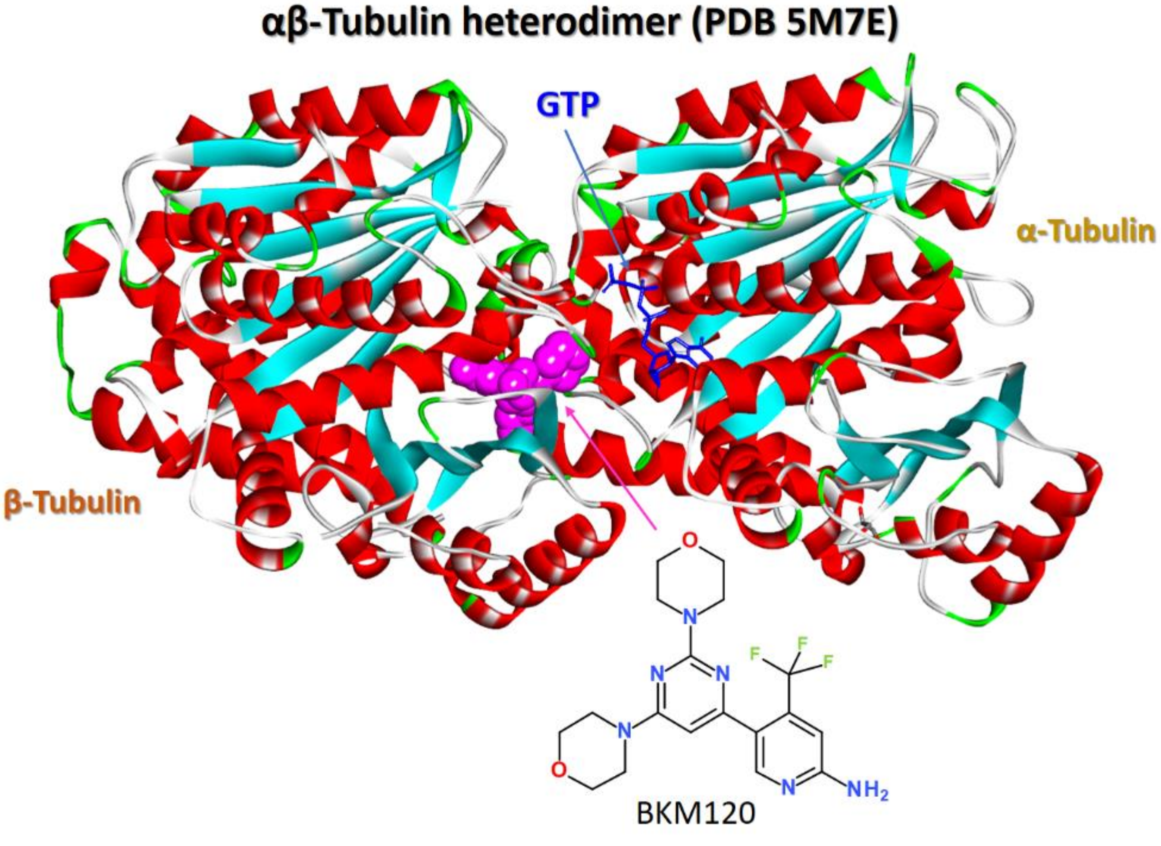

Fig. (S2). X-ray structure of the inhibitor BKM120 (Buparlisib) bound to αβ-tubulin heterodimer (PDB 5M7E) [2].

^{1}H and ^{13}C NMR spectra of **3, 5, 6, 8, 10a-k, 11** and **12**

3

^{1}H NMR (400 MHz, DMSO-d_6)

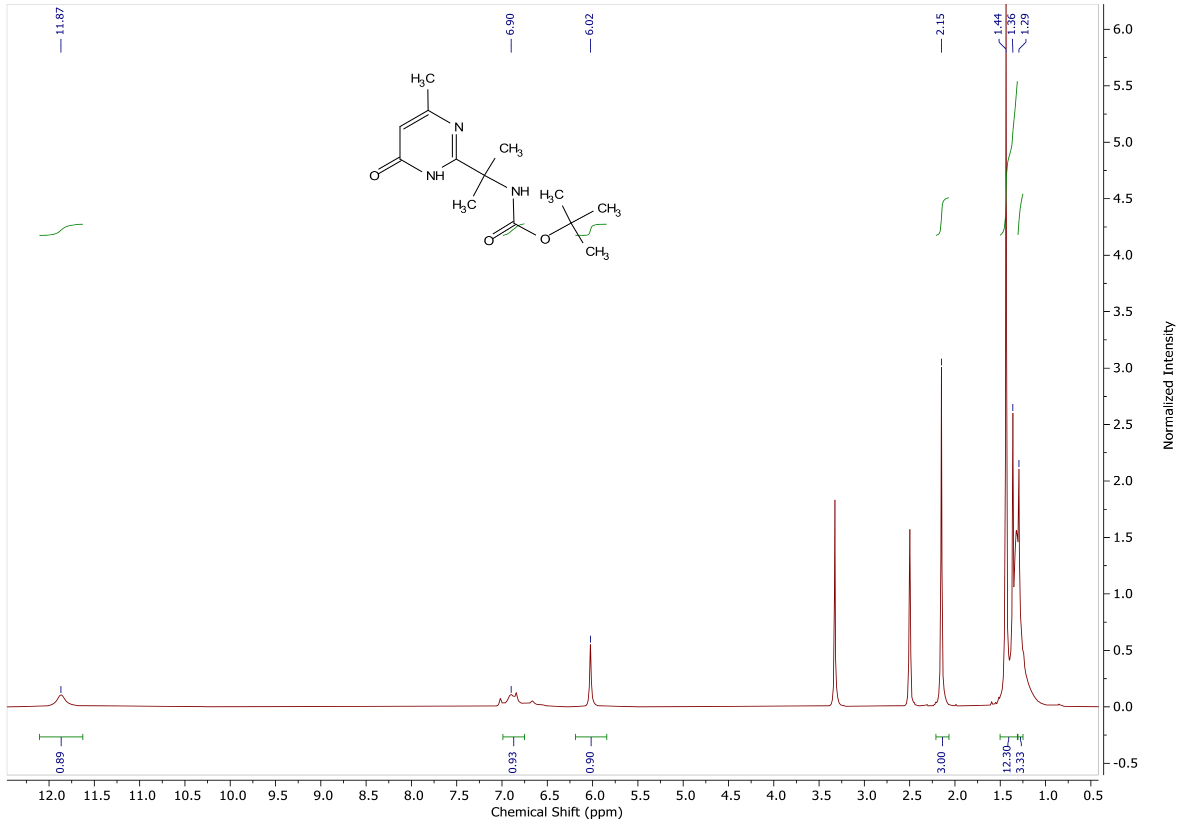

^{13}C NMR (126 MHz, DMSO-d_6)

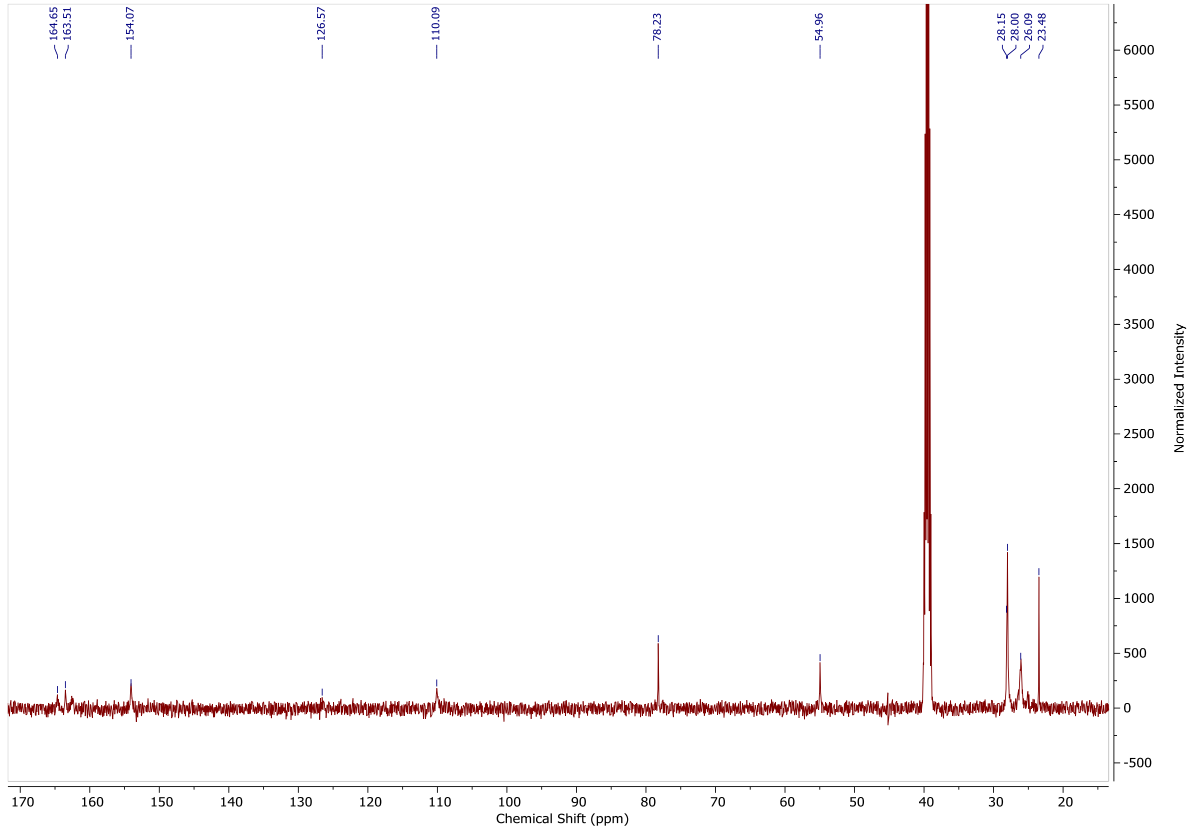

5

^{1}H NMR (400 MHz, DMSO-d_6)

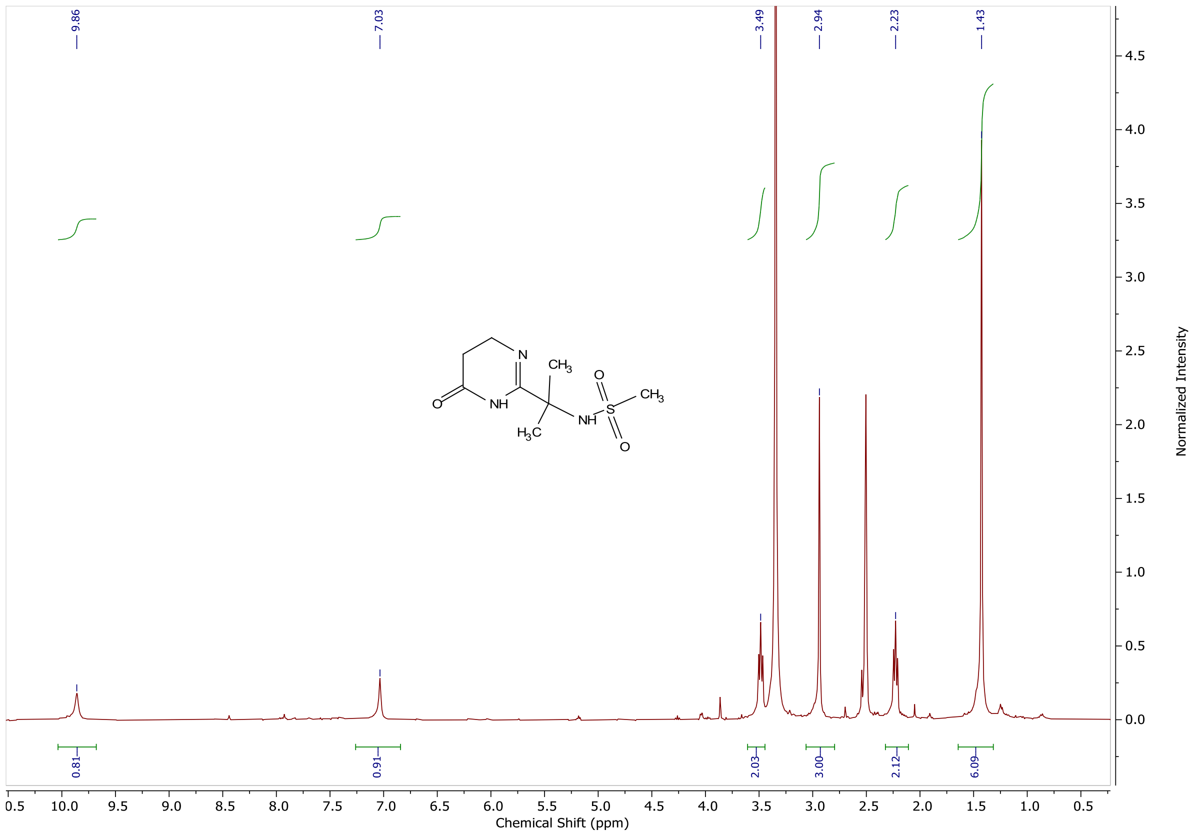

^{13}C NMR (126 MHz, DMSO-d_6)

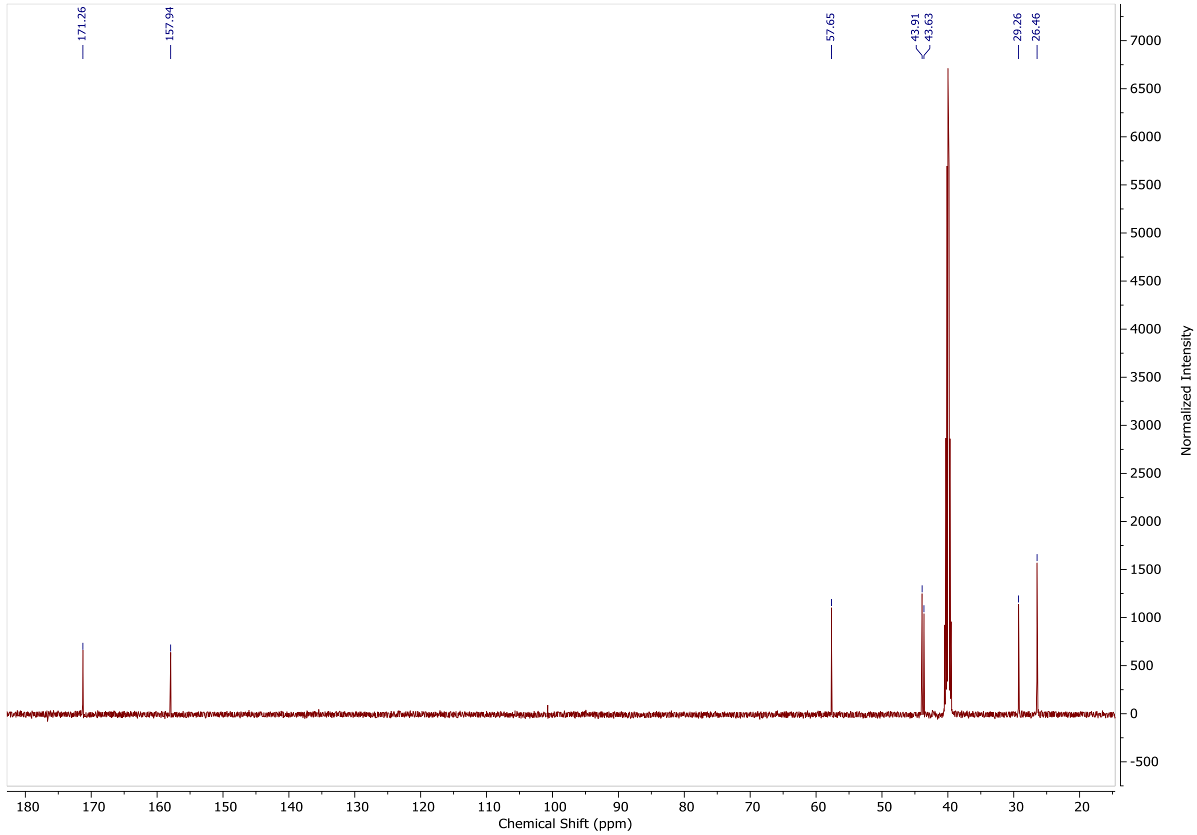

6

^{1}H NMR (400 MHz, DMSO-d_6)

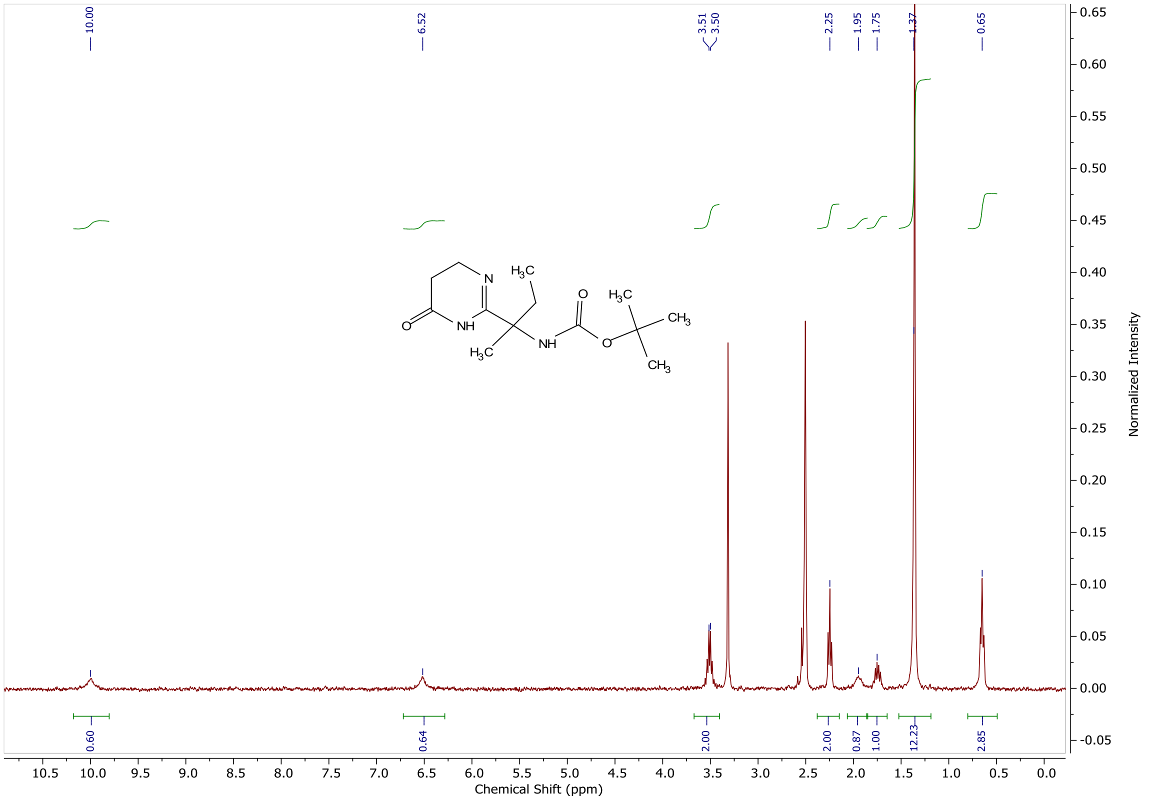

^{13}C NMR (126 MHz, DMSO-d_6)

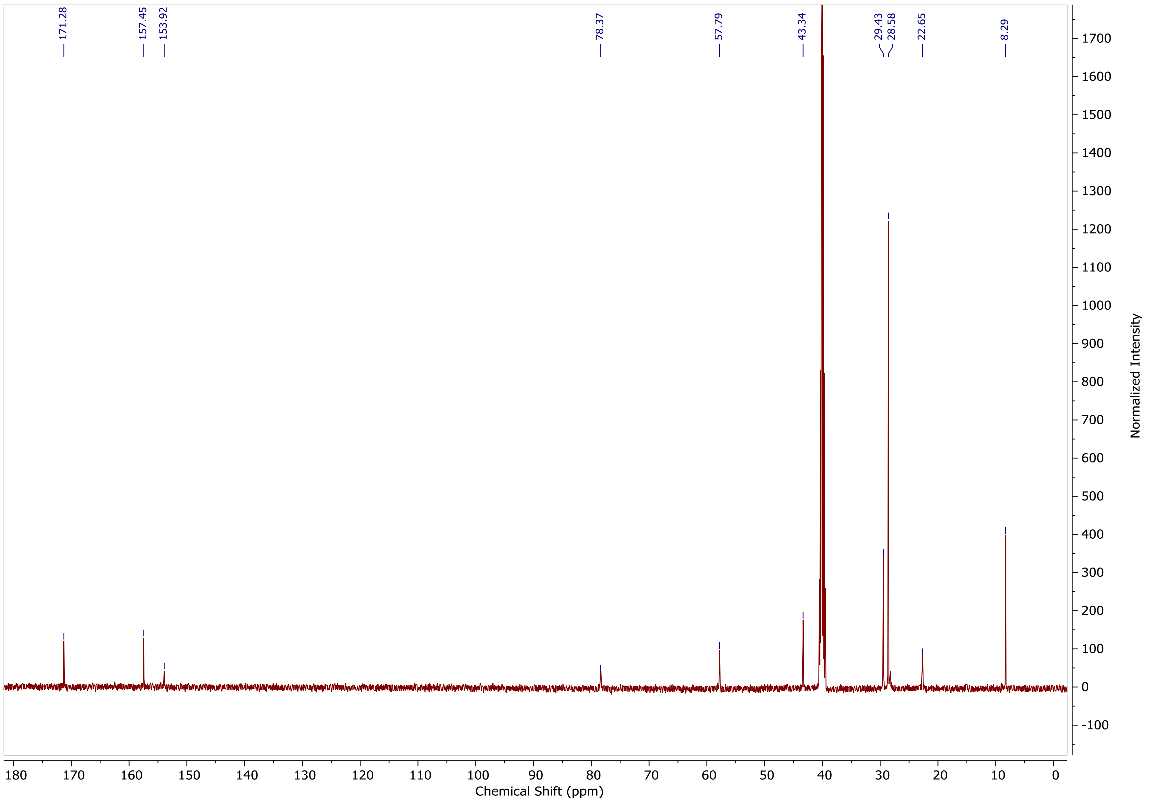

8

^{1}H NMR (400 MHz, DMSO-d_6)

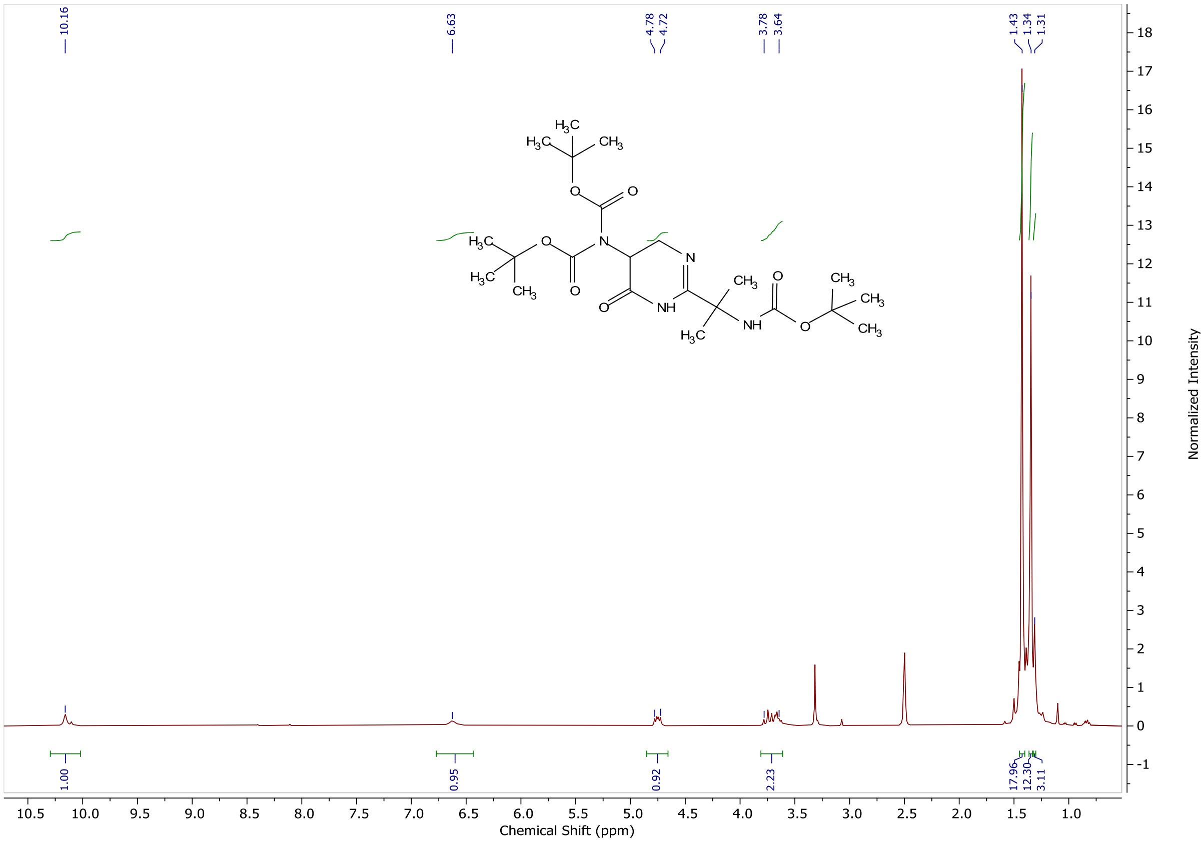

^{13}C NMR (126 MHz, DMSO-d_6)

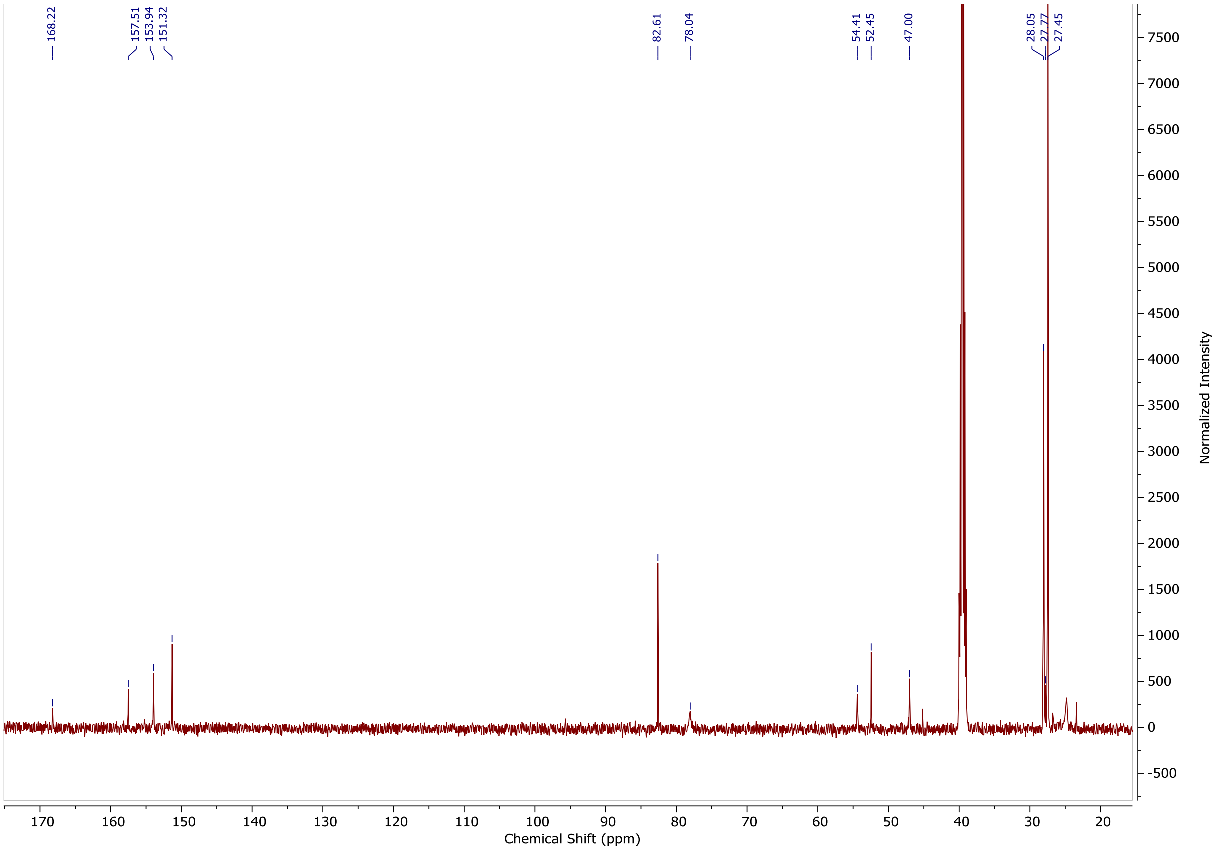

10a

^{1}H NMR (400 MHz, DMSO-d_6)

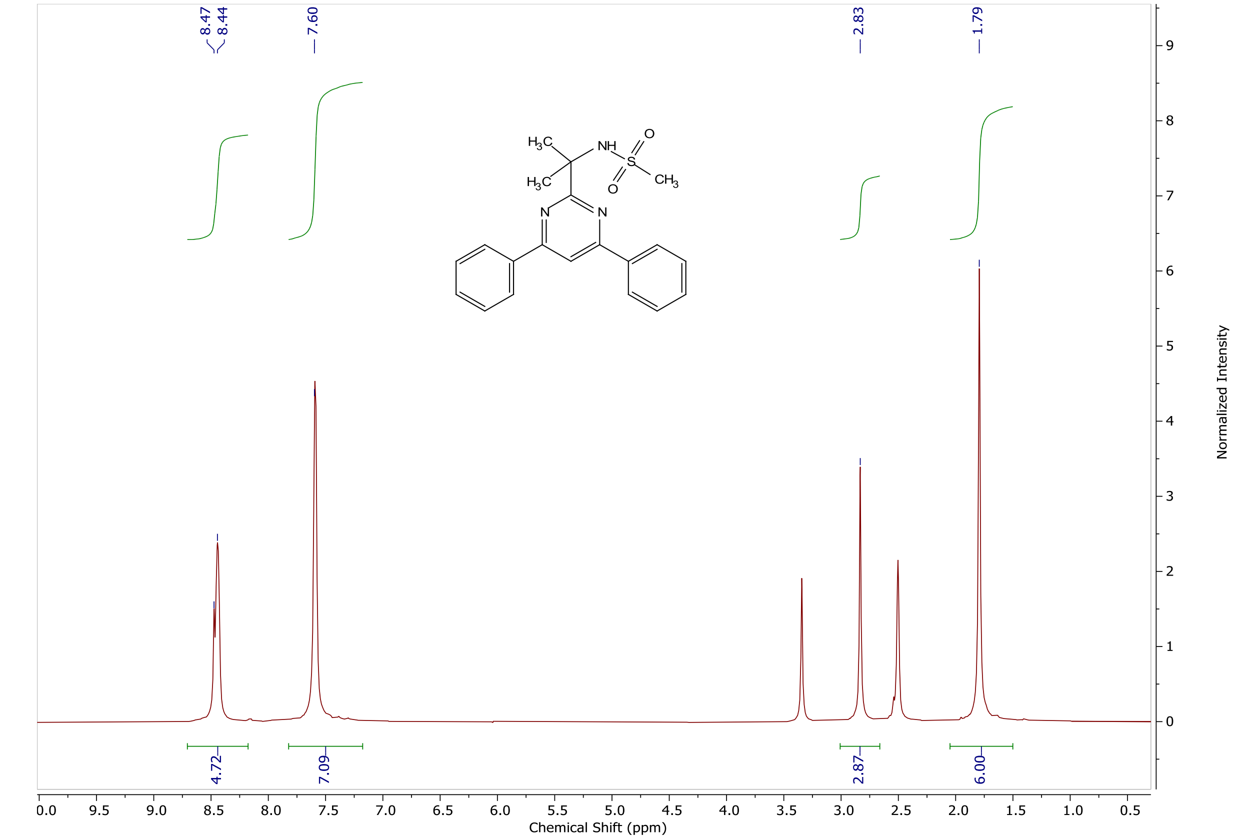

^{13}C NMR (126 MHz, DMSO-d_6)

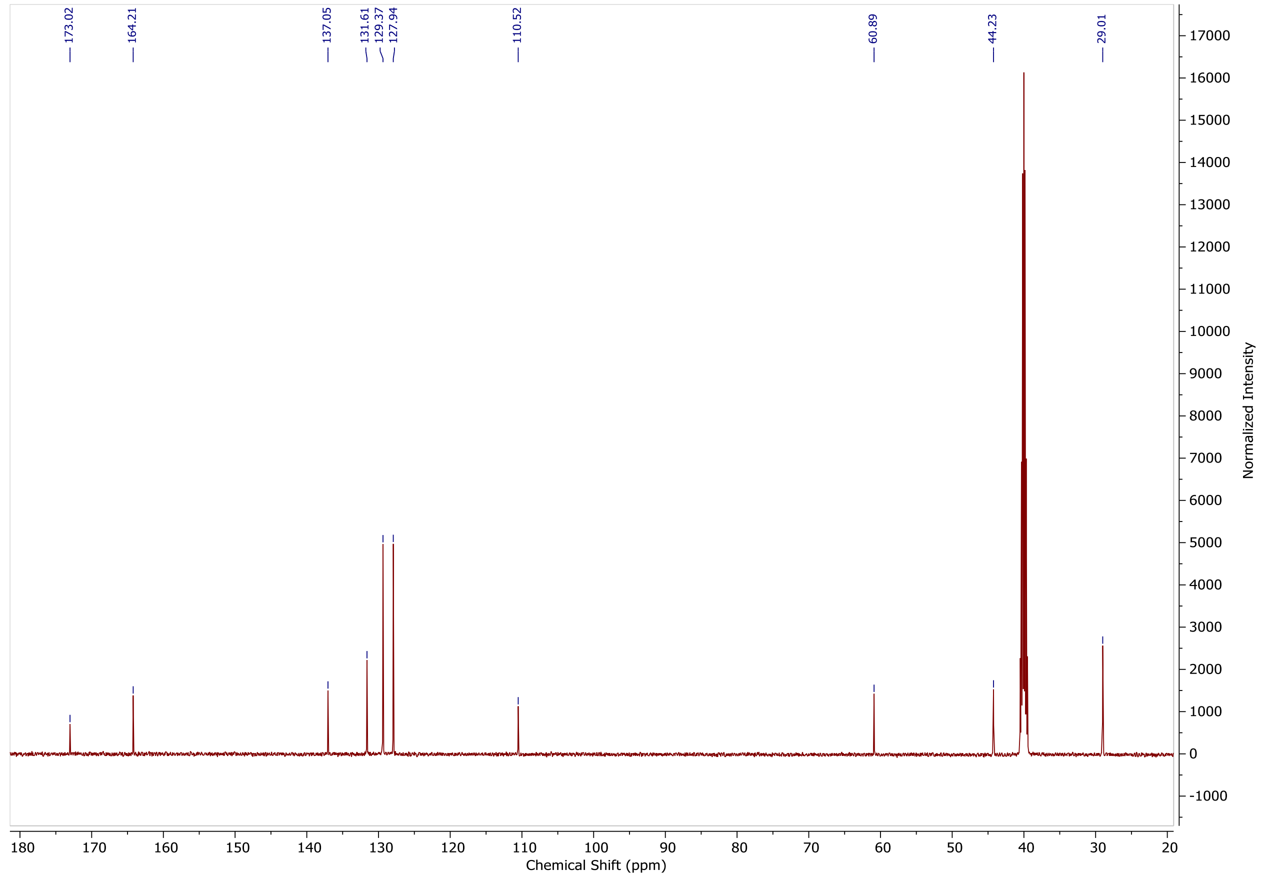

10b

^{1}H NMR (400 MHz, DMSO-d_6)

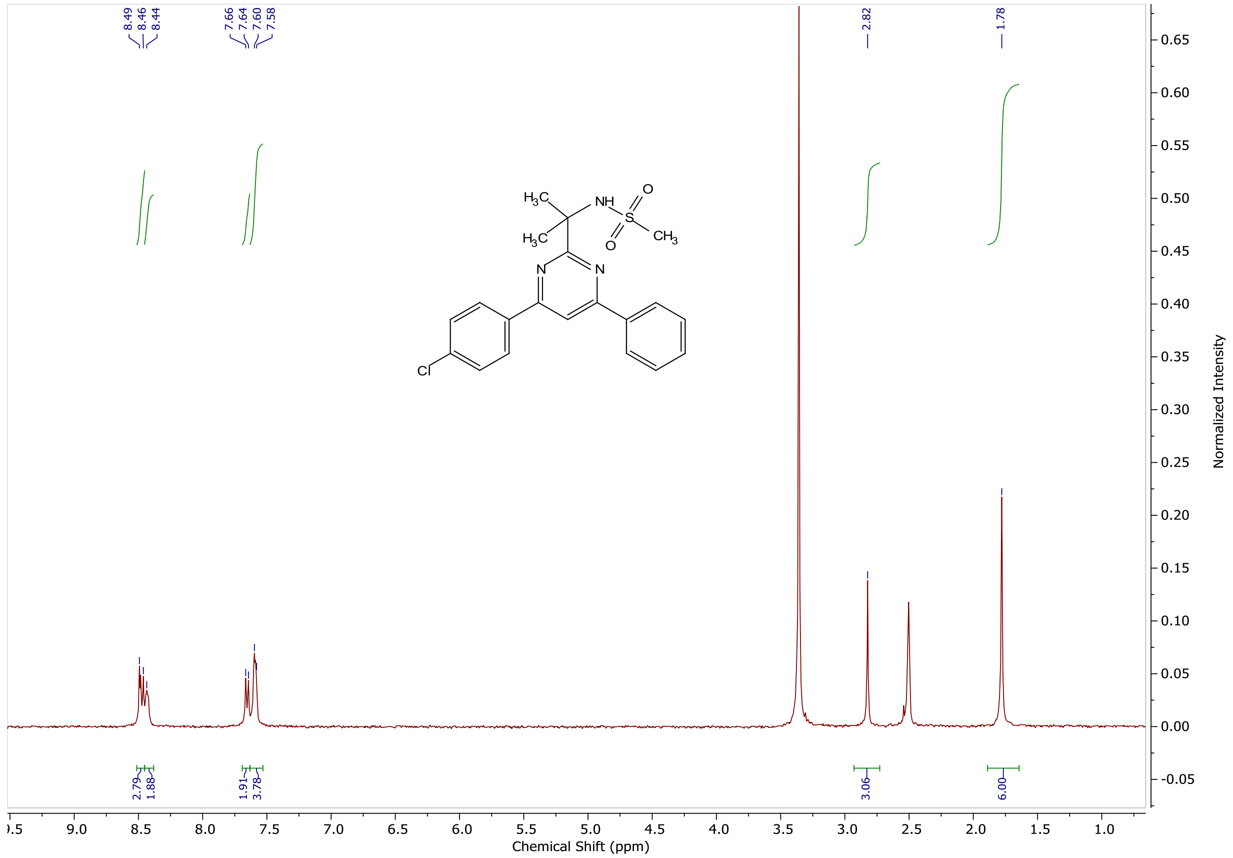

^{13}C NMR (126 MHz, DMSO-d_6)

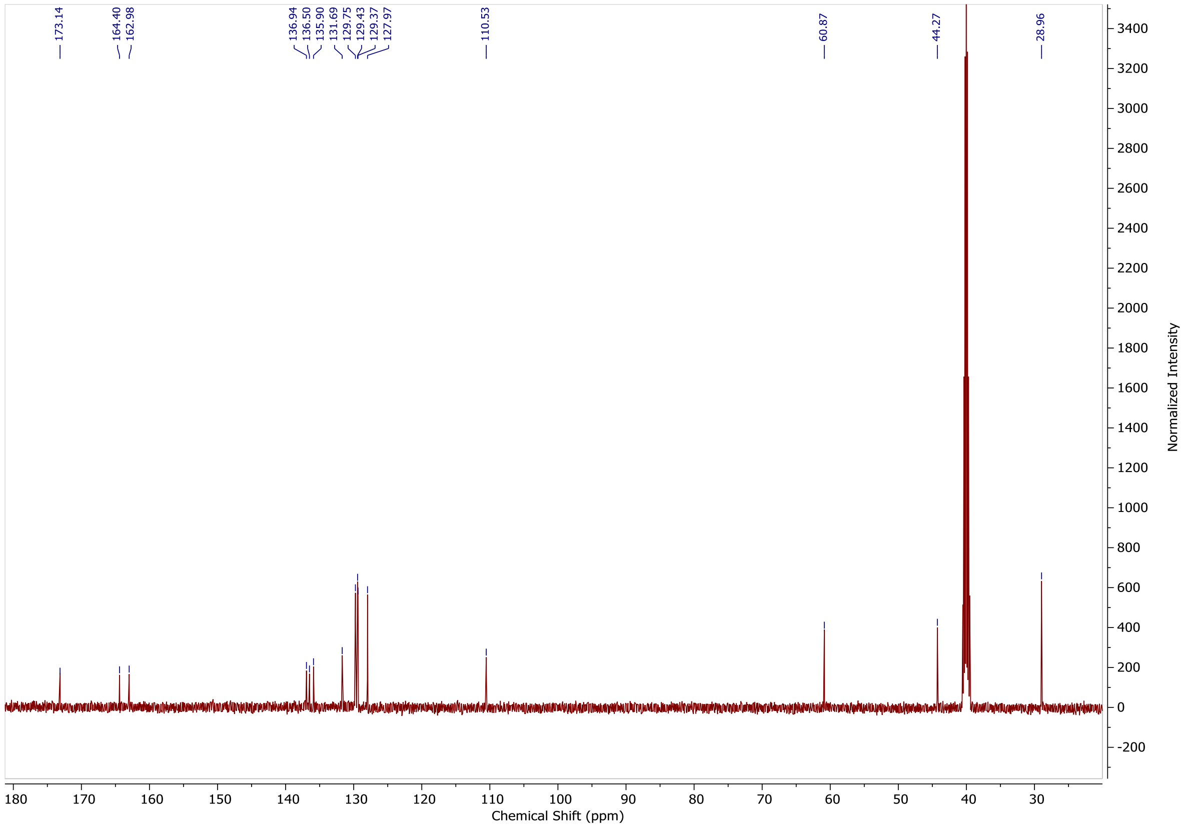

10c

^{1}H NMR (400 MHz, DMSO-d_6)

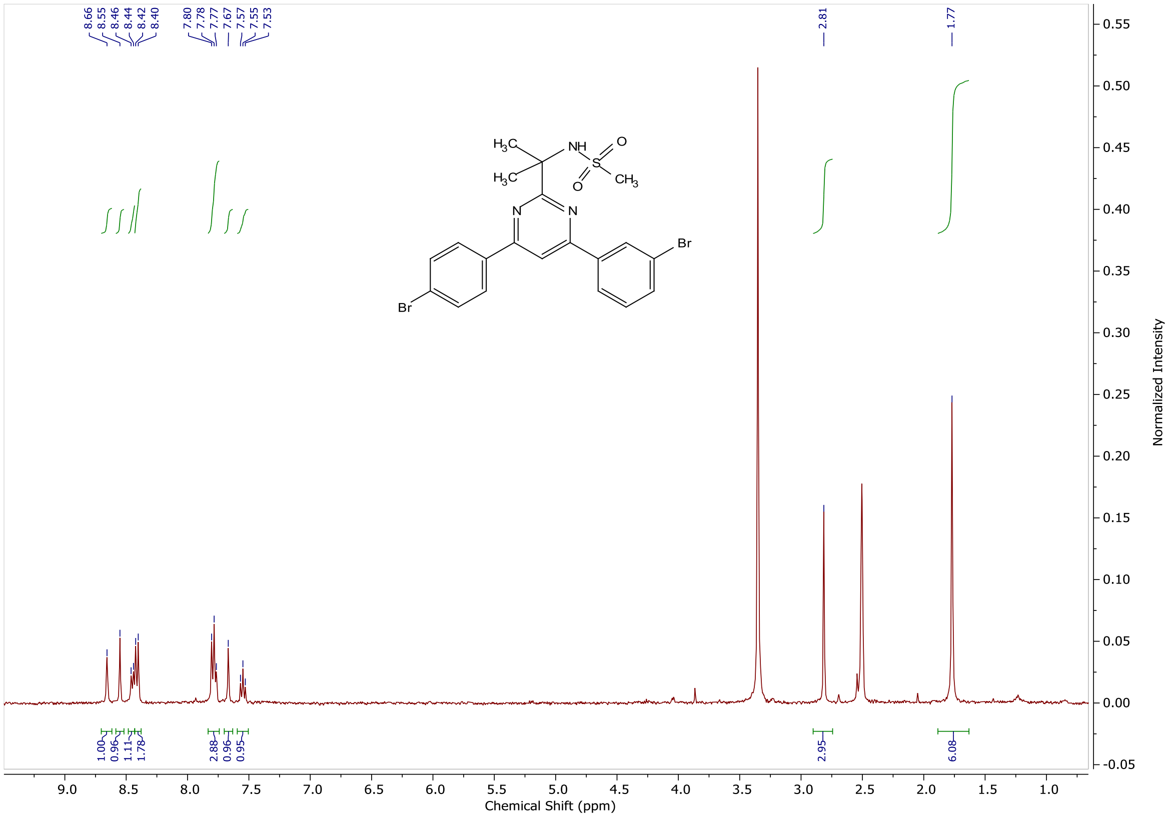

^{13}C NMR (126 MHz, DMSO-d_6)

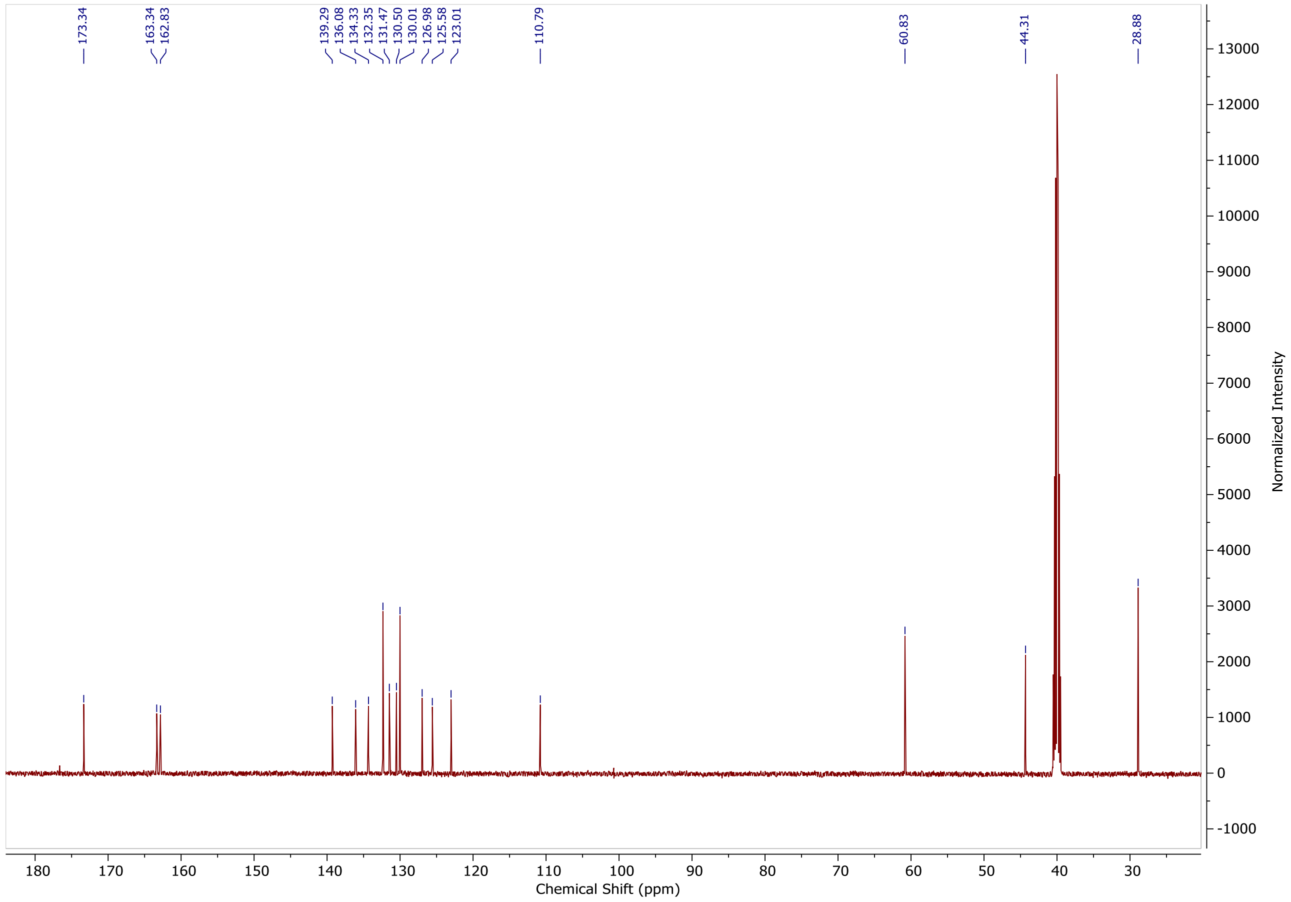

10d

^{1}H NMR (400 MHz, DMSO-d_6)

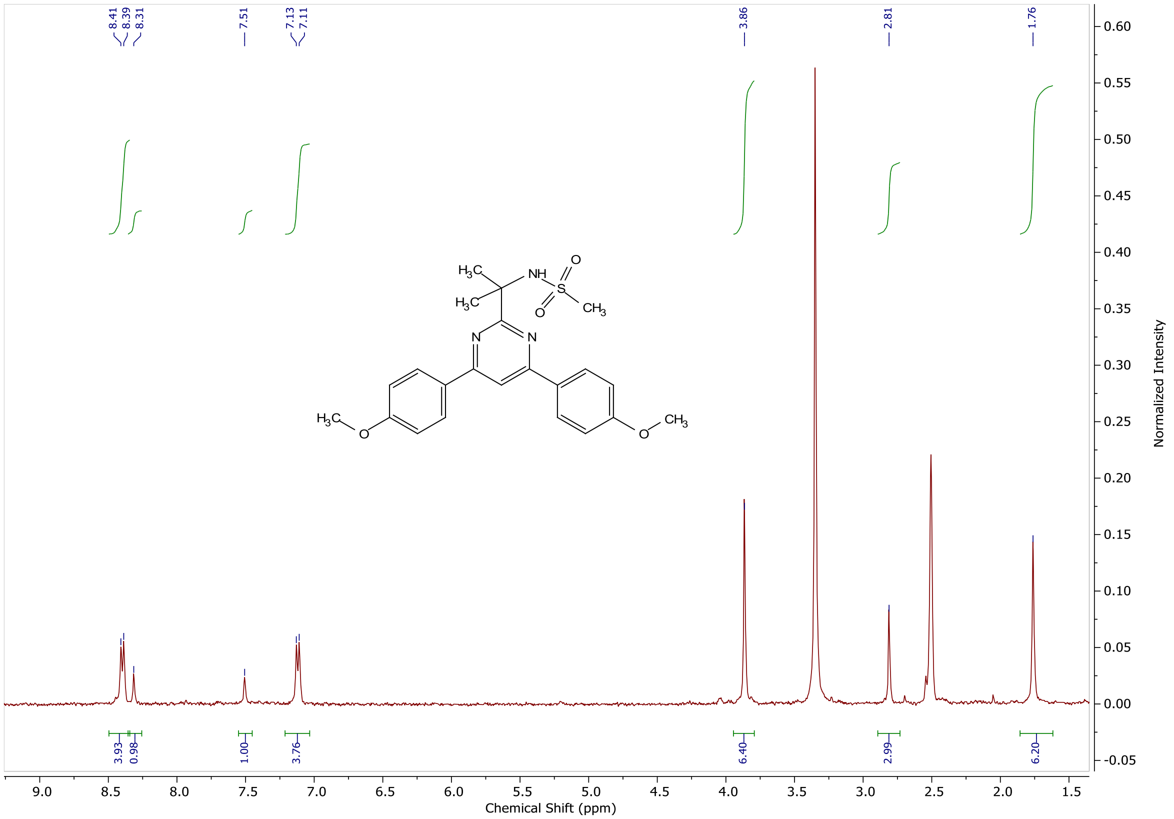

^{13}C NMR (126 MHz, DMSO-d_6)

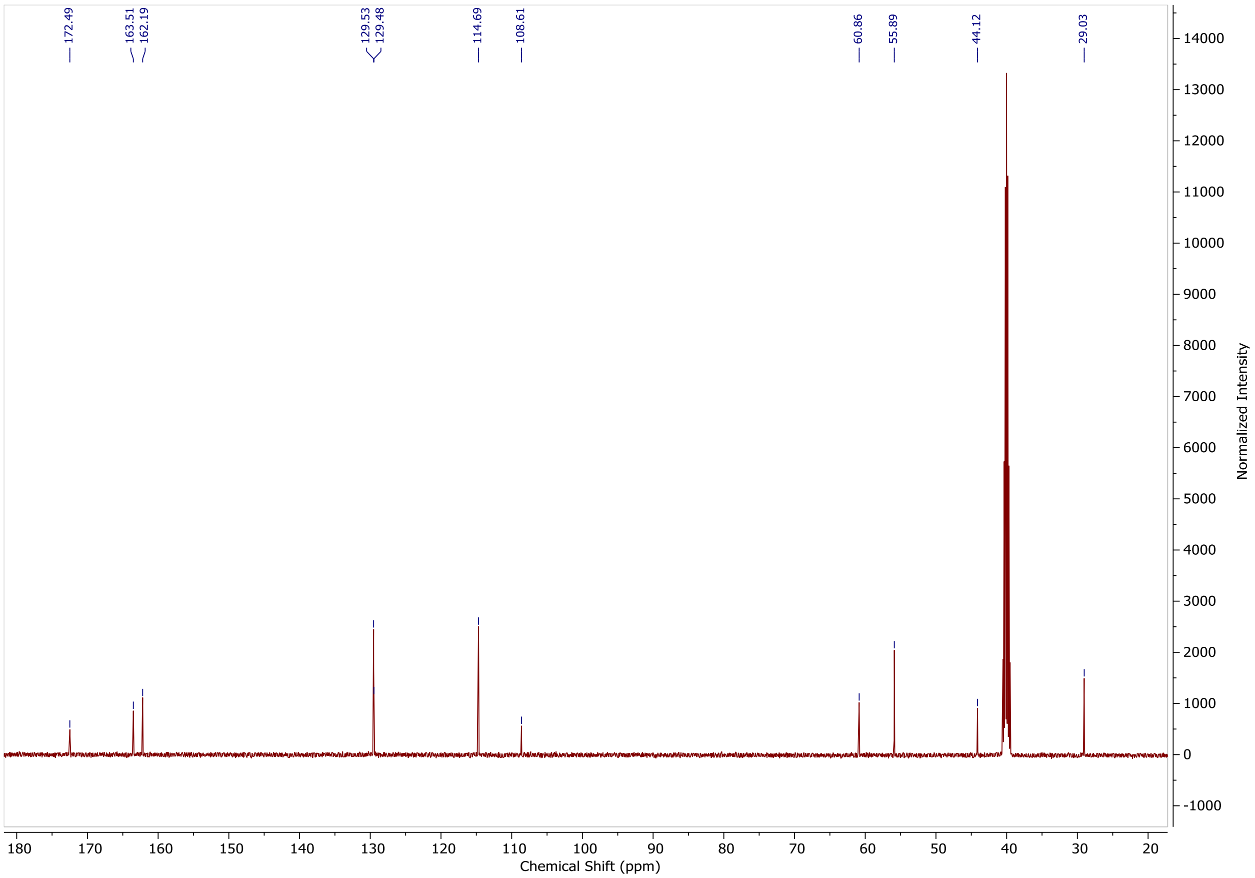

10e

^{1}H NMR (400 MHz, DMSO-d_6)

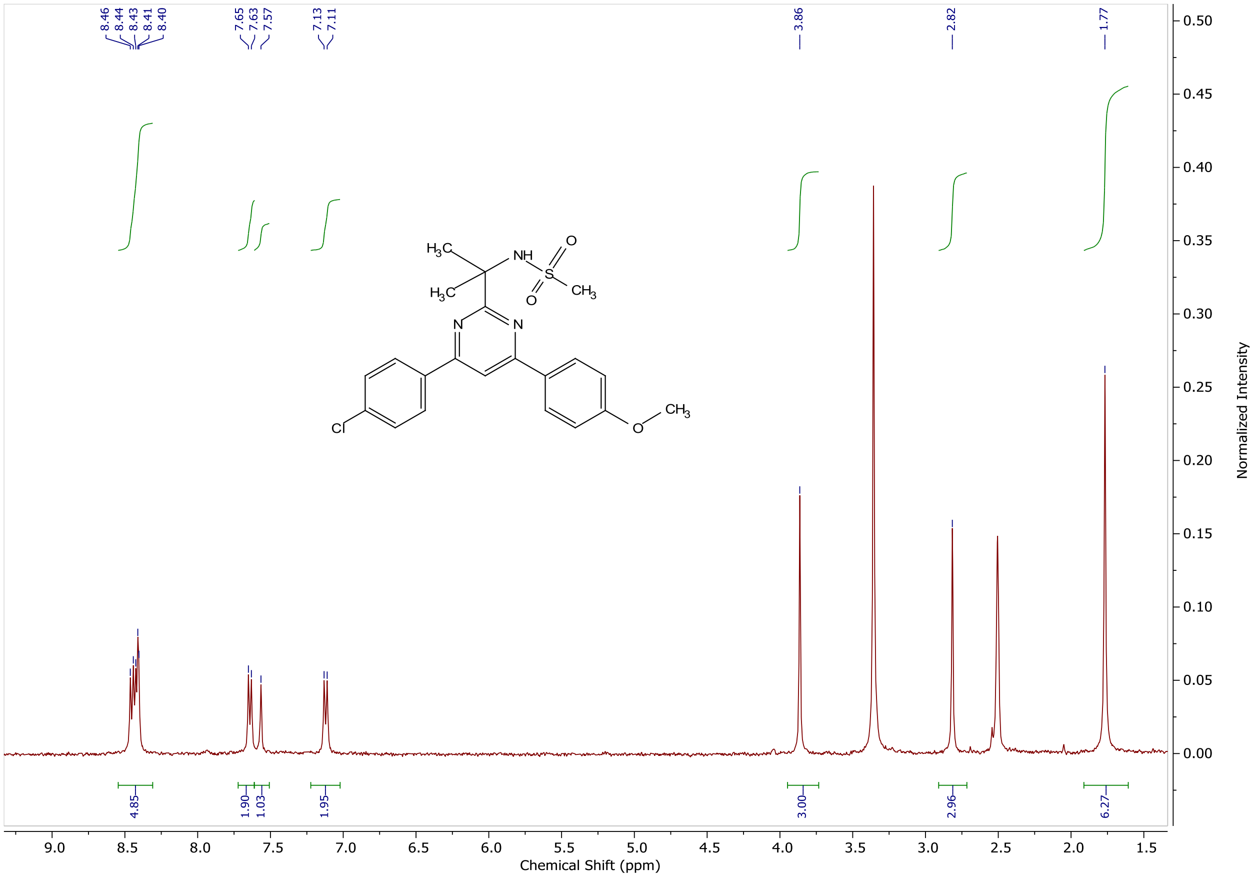

^{13}C NMR (126 MHz, DMSO-d_6)

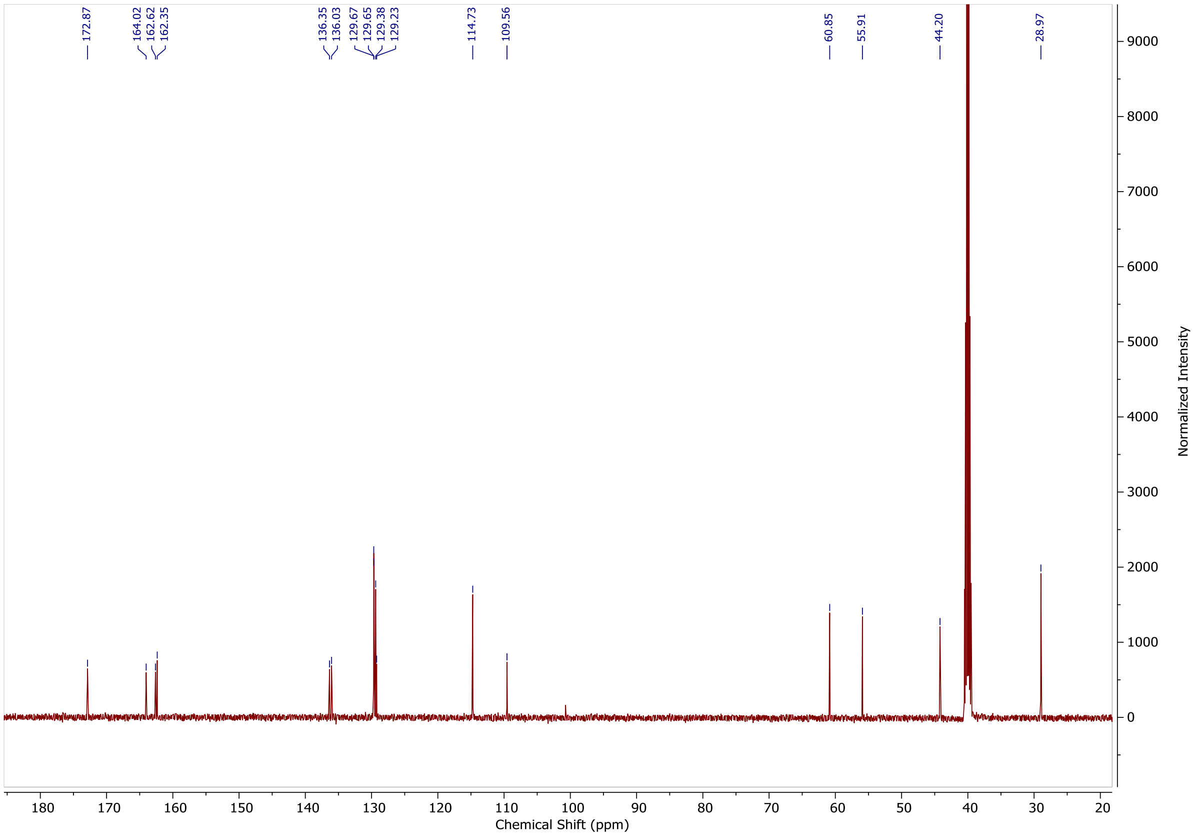

10f

^{1}H NMR (400 MHz, DMSO-d_6)

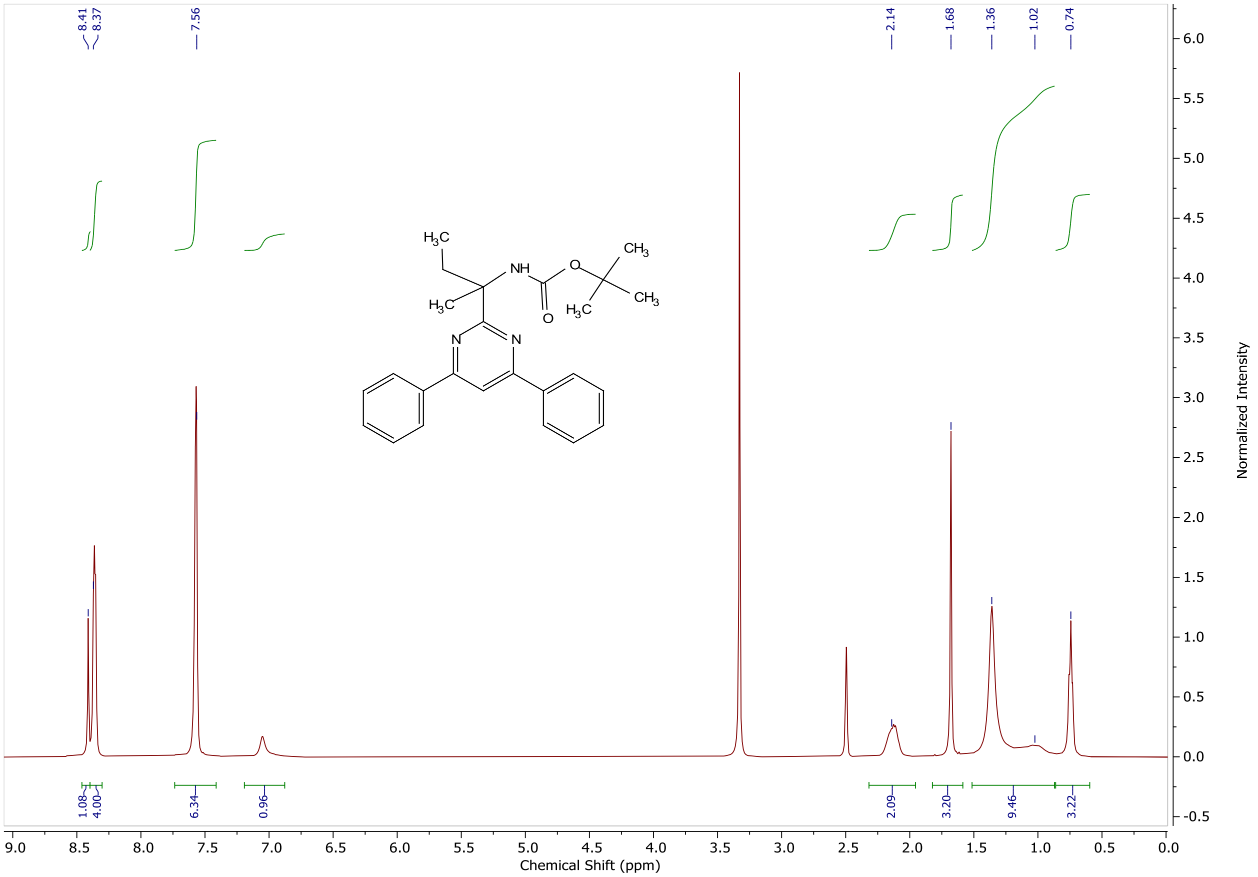

^{13}C NMR (126 MHz, DMSO-d_6)

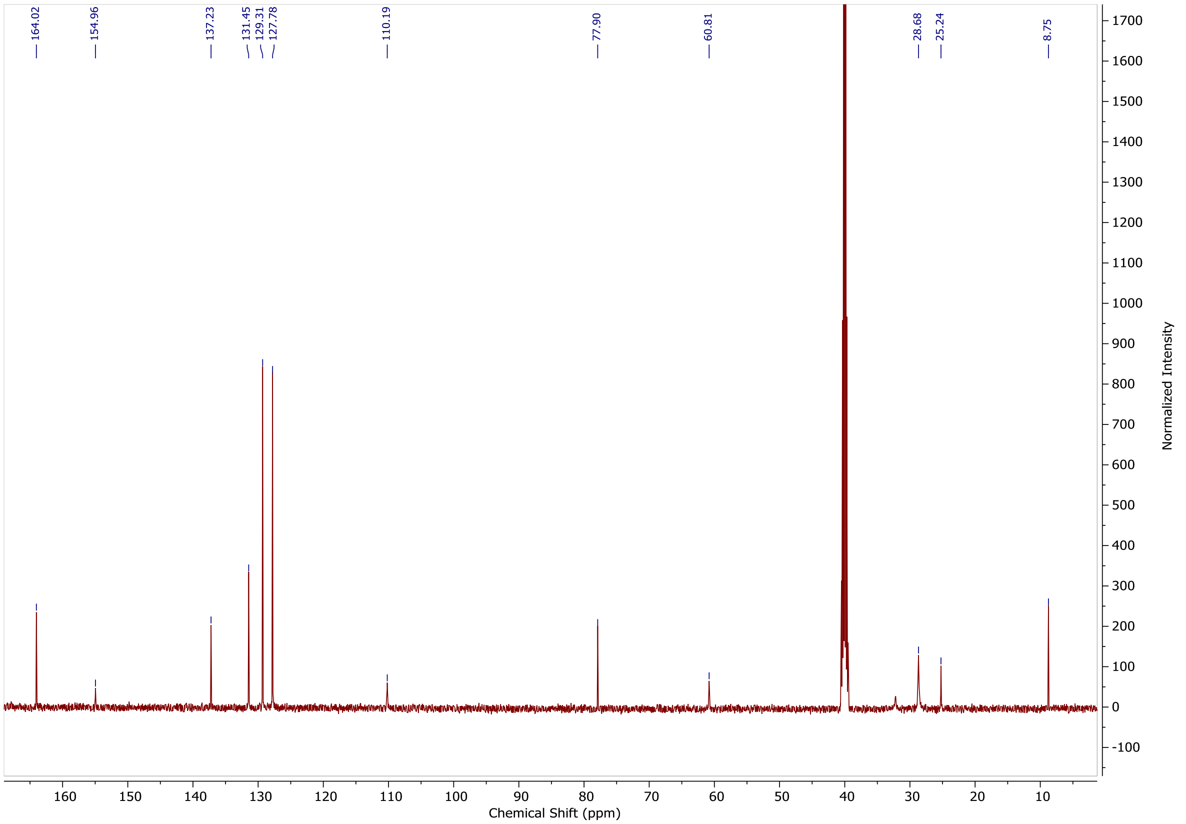

10g

^{1}H NMR (500 MHz, DMSO-d_6)

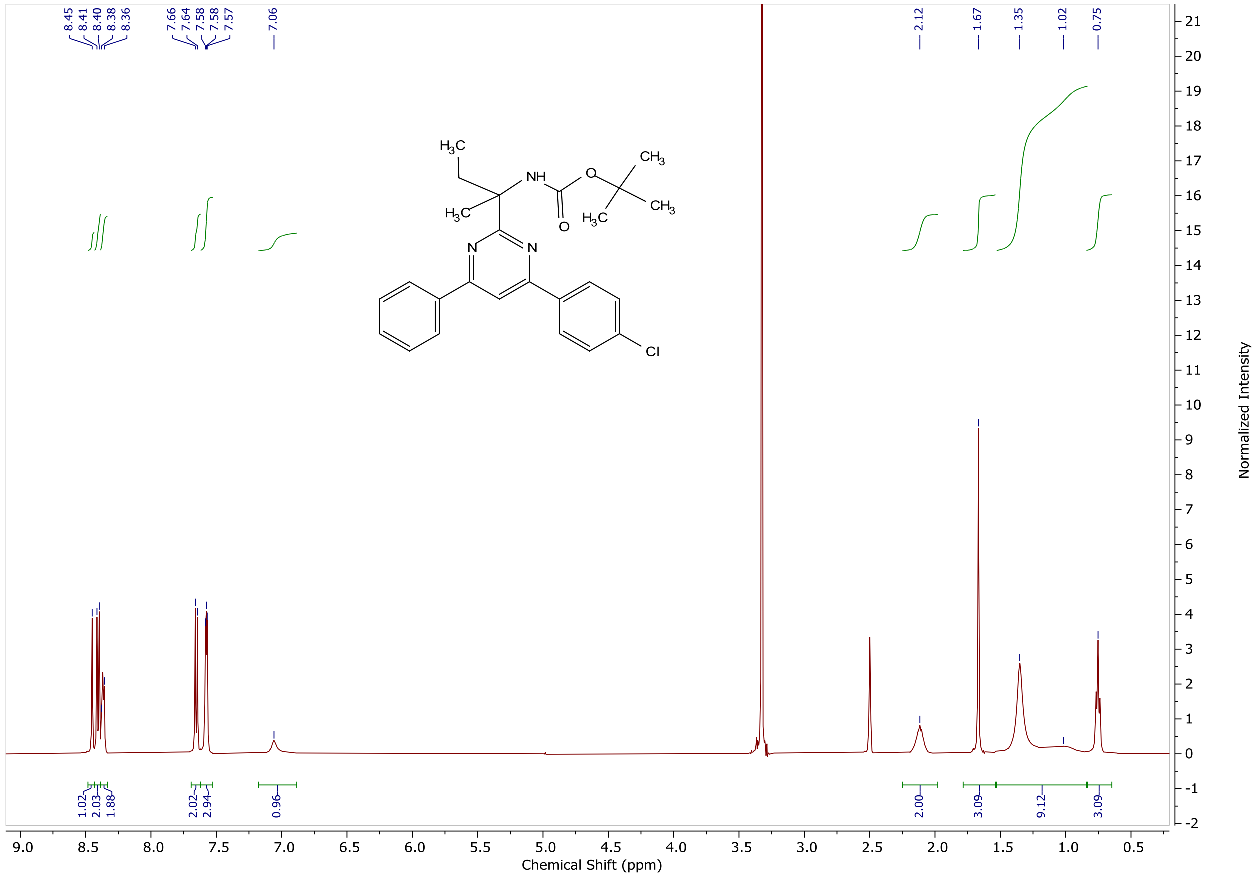

^{13}C NMR (126 MHz, DMSO-d_6)

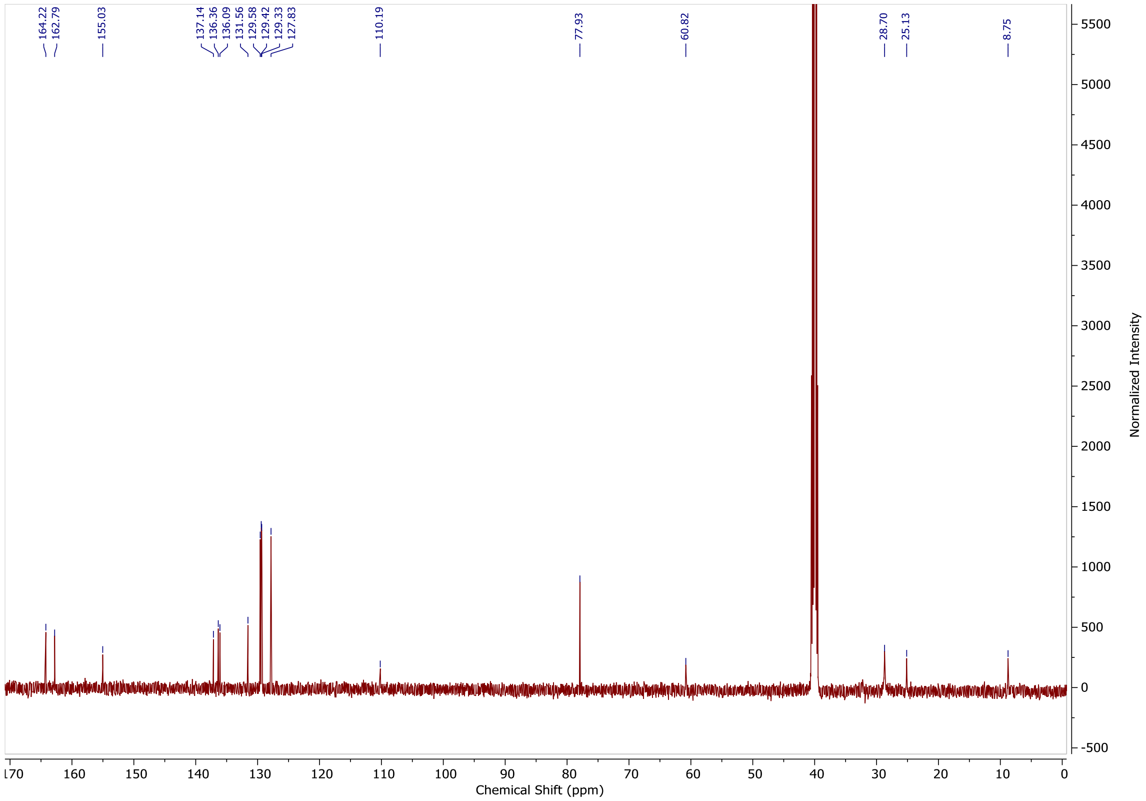

10h

^{1}H NMR (500 MHz, DMSO-d_6)

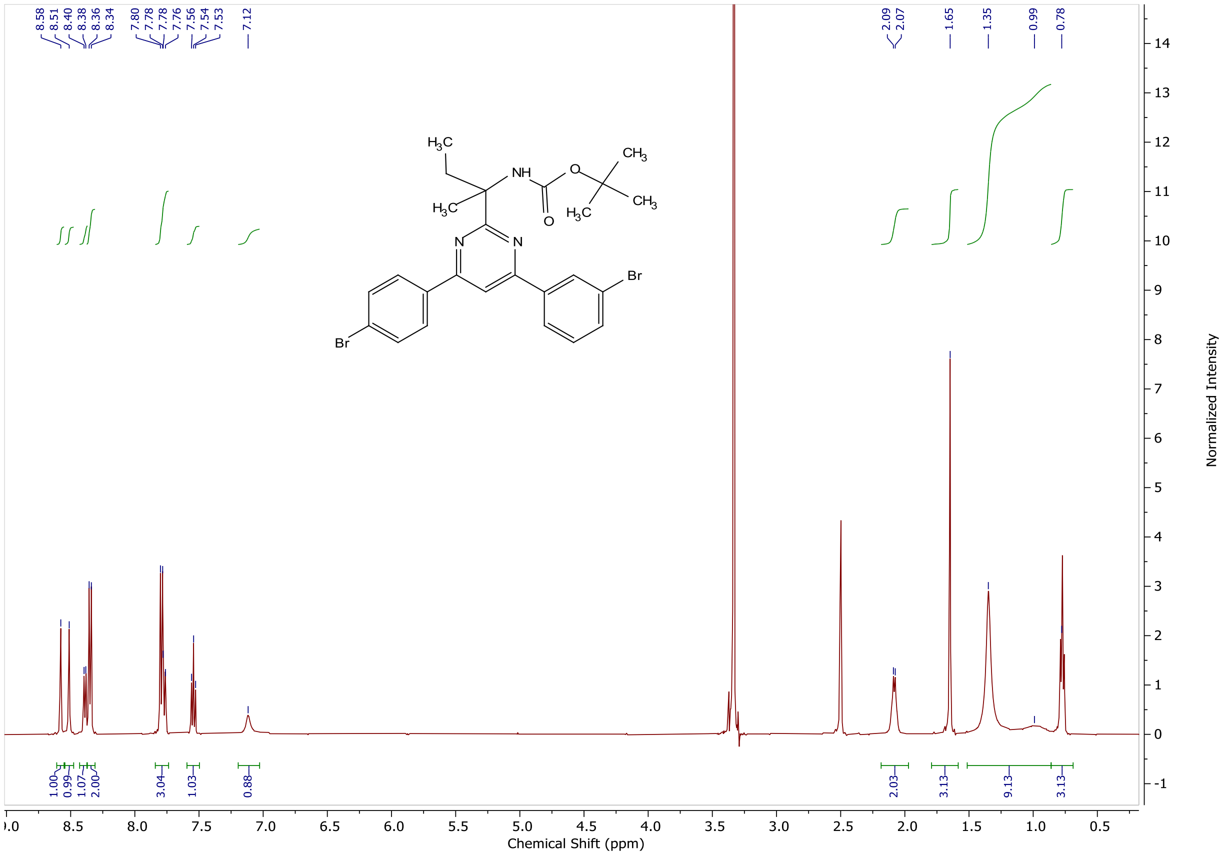

^{13}C NMR (126 MHz, DMSO-d_6)

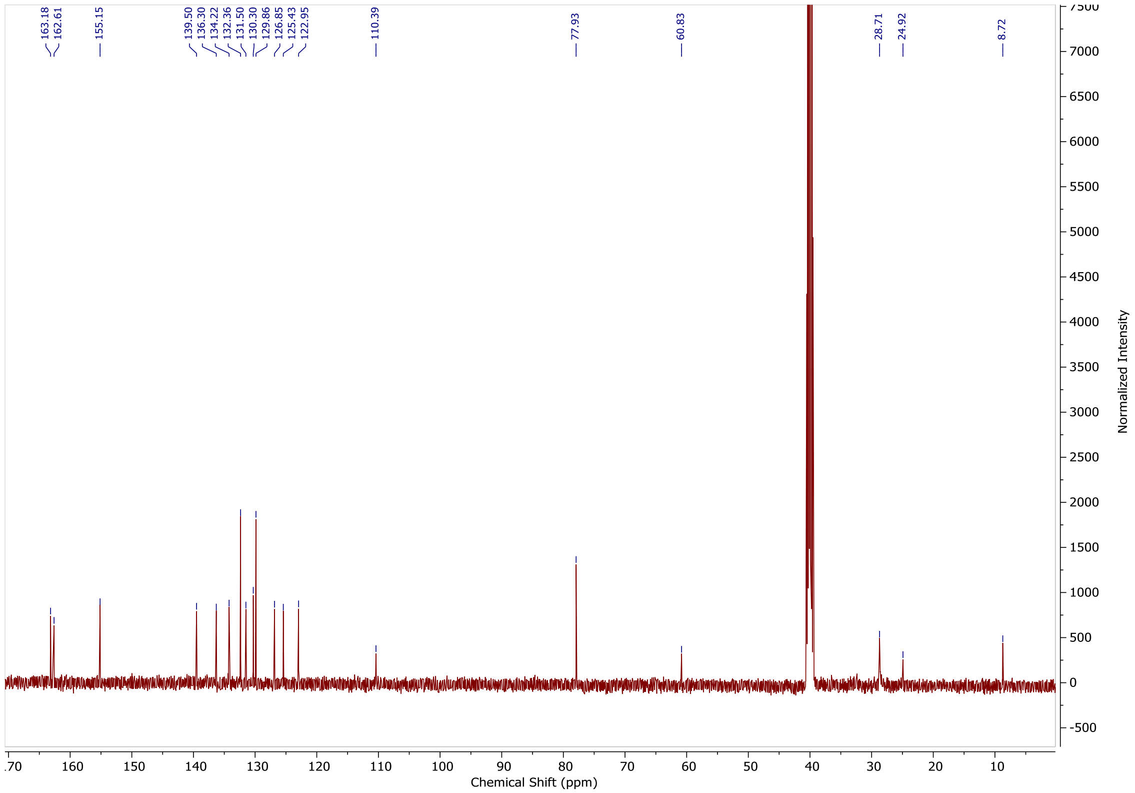

10i

^{1}H NMR (500 MHz, DMSO-d_6)

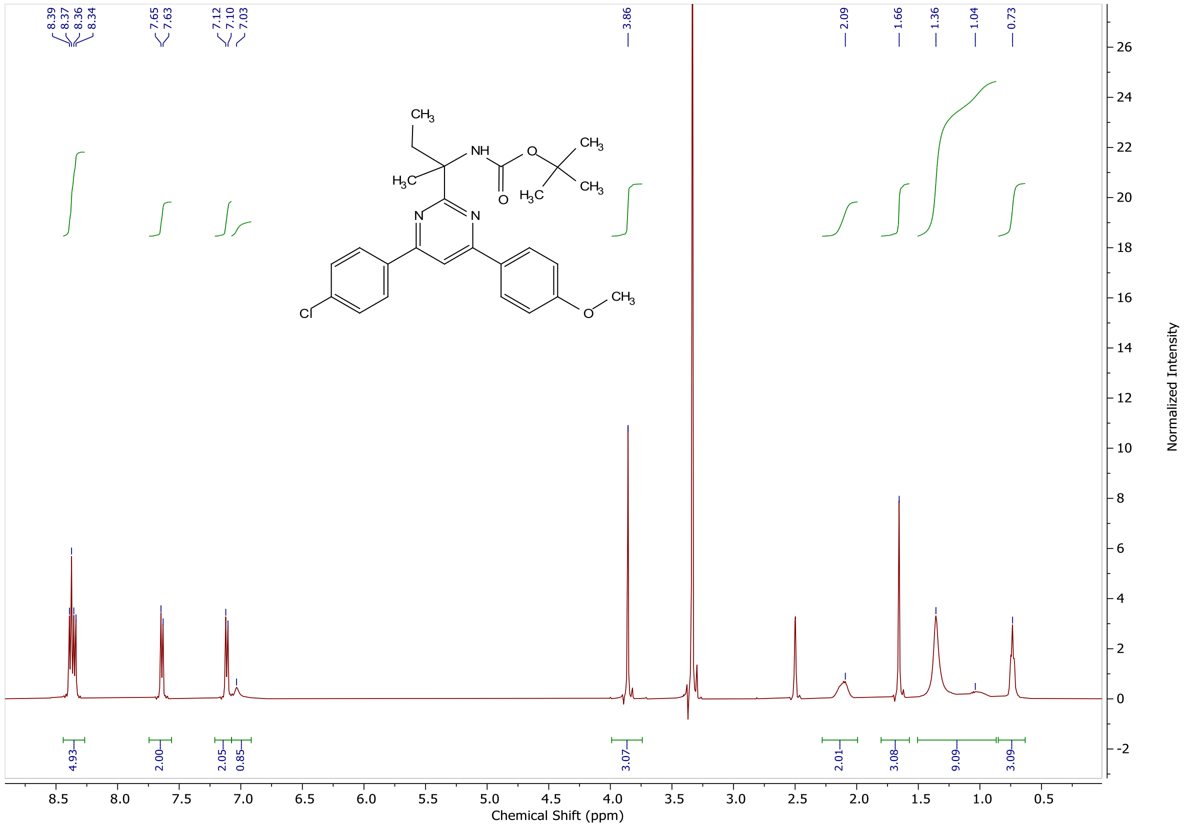

^{13}C NMR (126 MHz, DMSO-d_6)

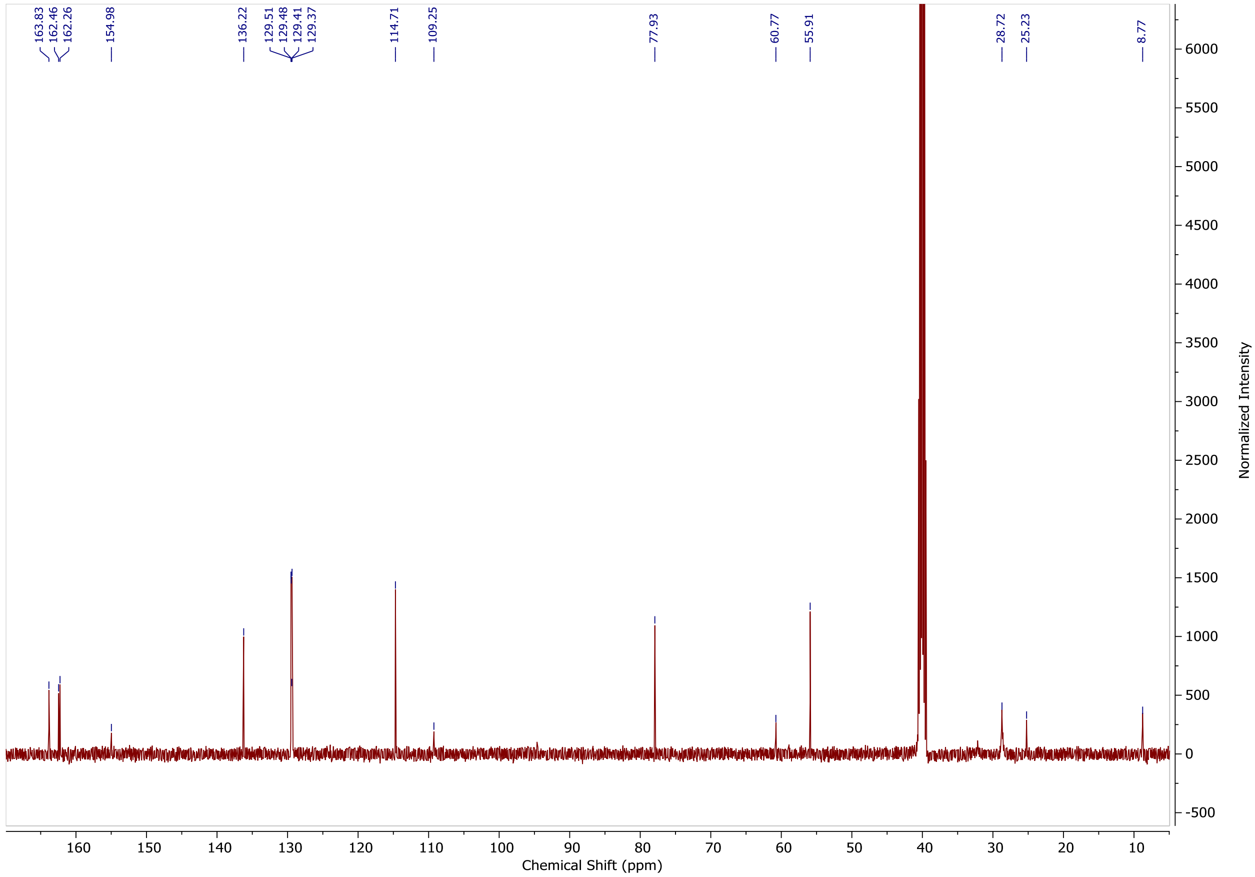

10k

^{1}H NMR (500 MHz, DMSO-d_6)

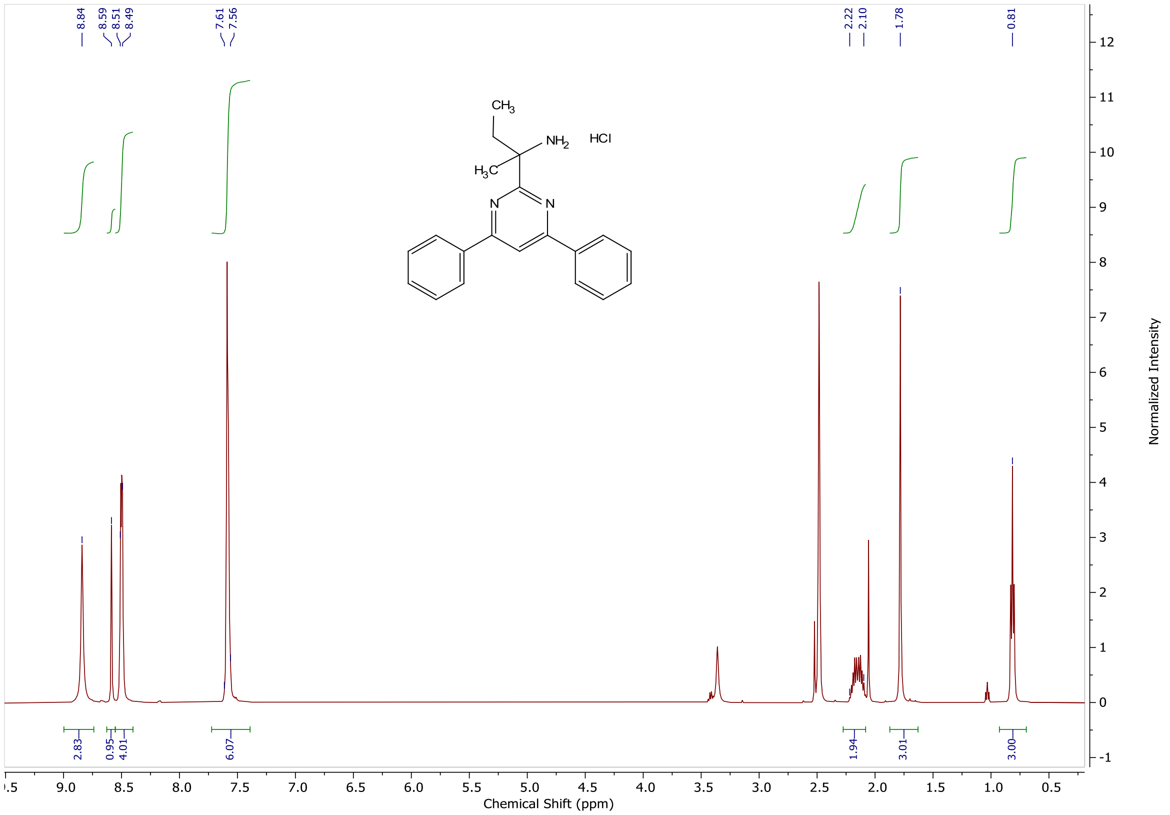

^{13}C NMR (126 MHz, DMSO-d_6)

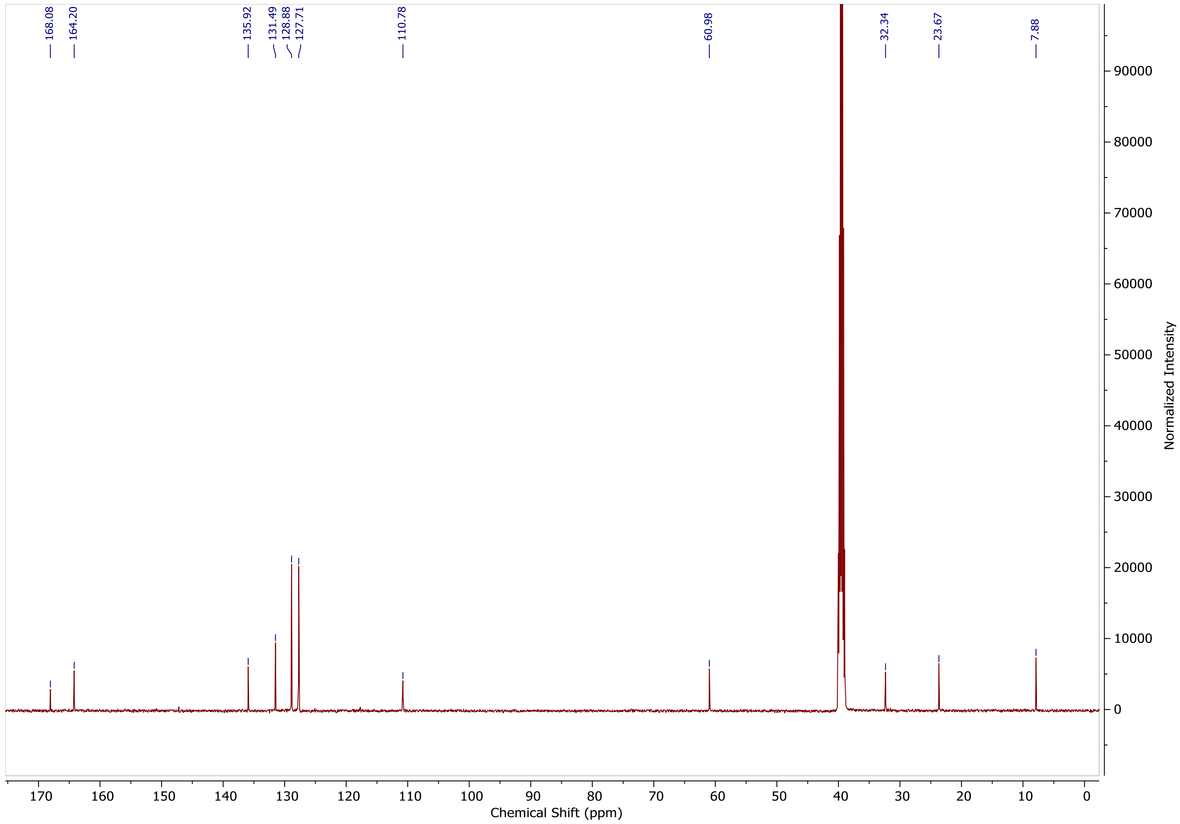

11

^{1}H NMR (500 MHz, DMSO-d_6)

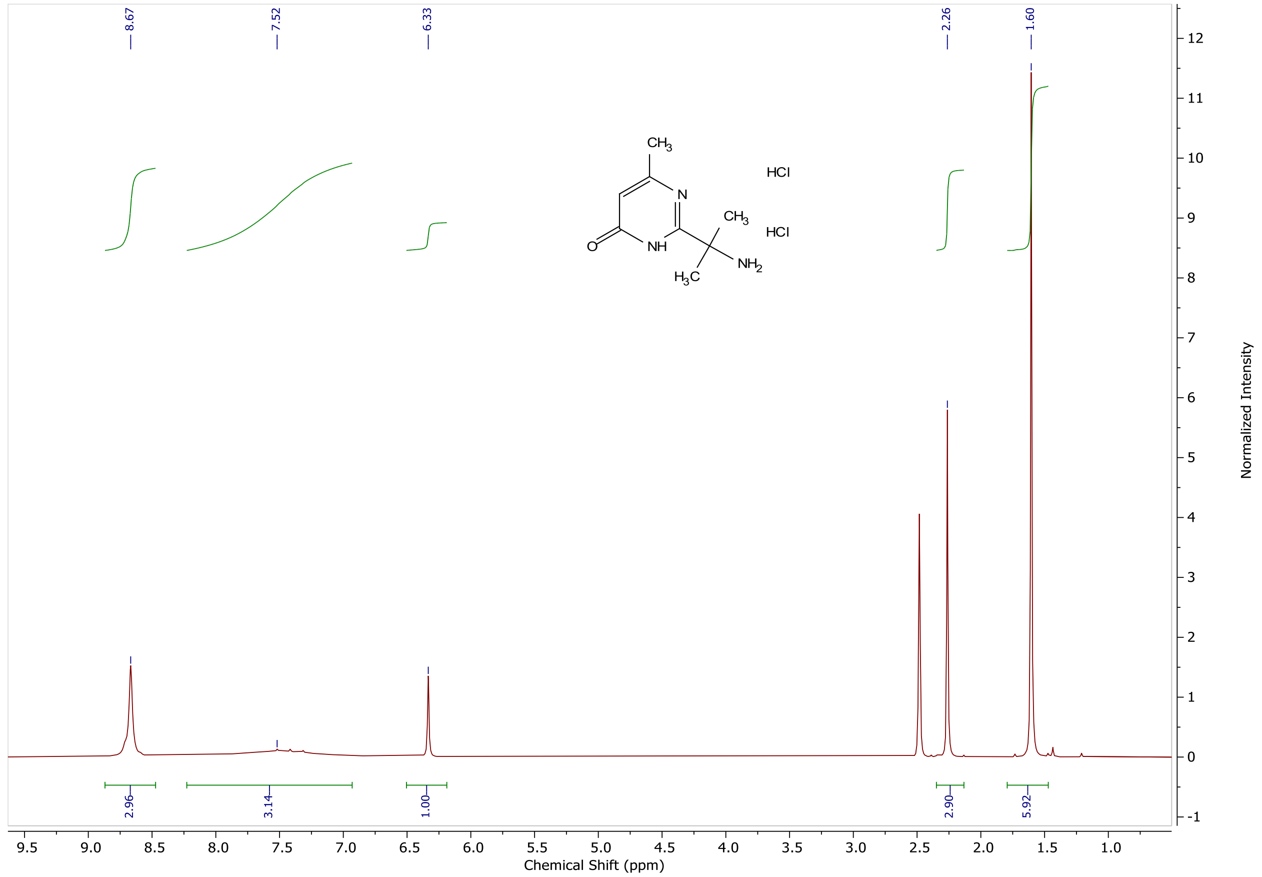

^{13}C NMR (126 MHz, DMSO-d_6)

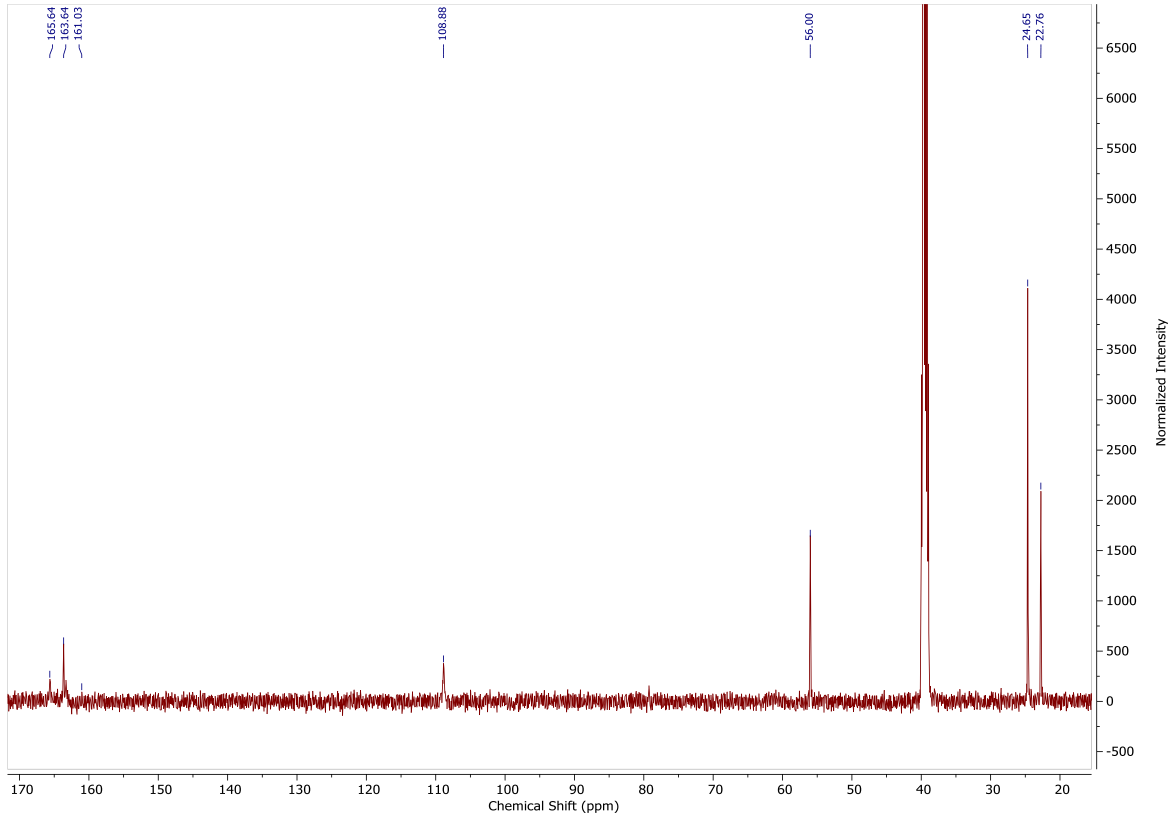

12

^{1}H NMR (400 MHz, DMSO-d_6)

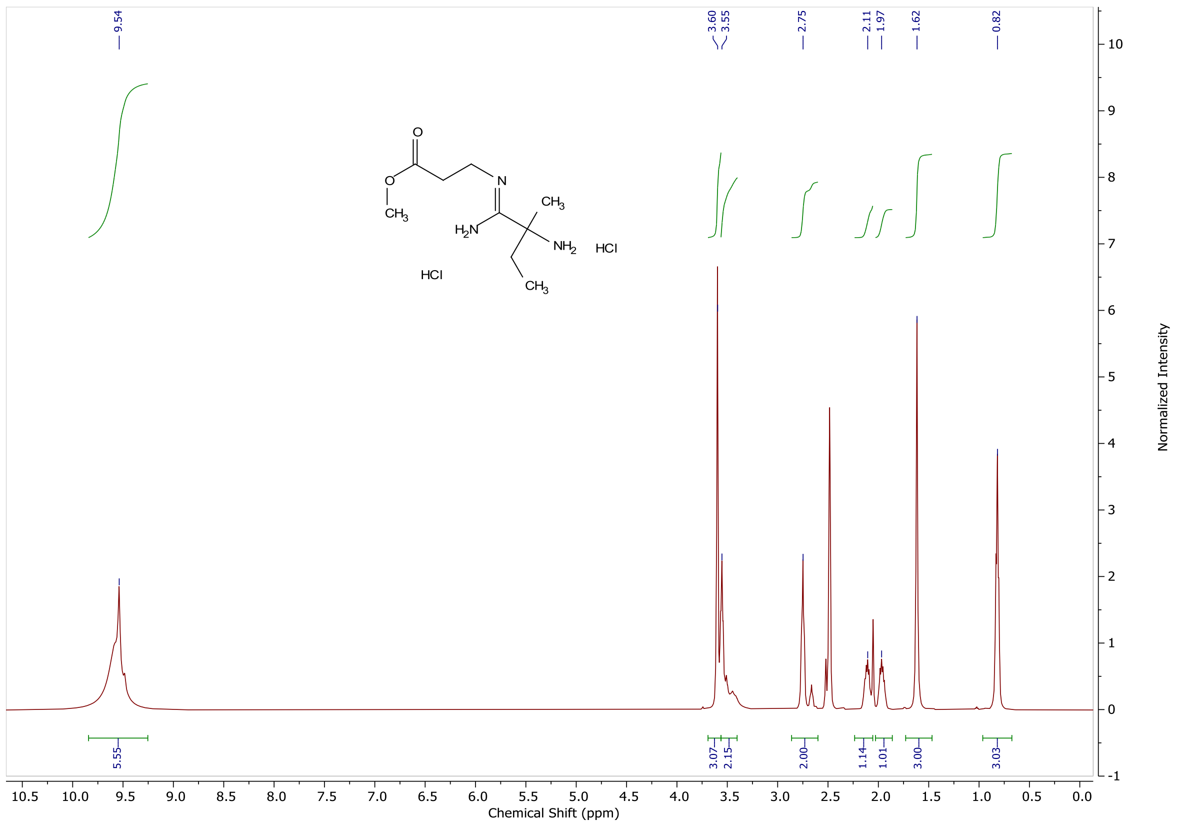

^{13}C NMR (126 MHz, DMSO-d_6)

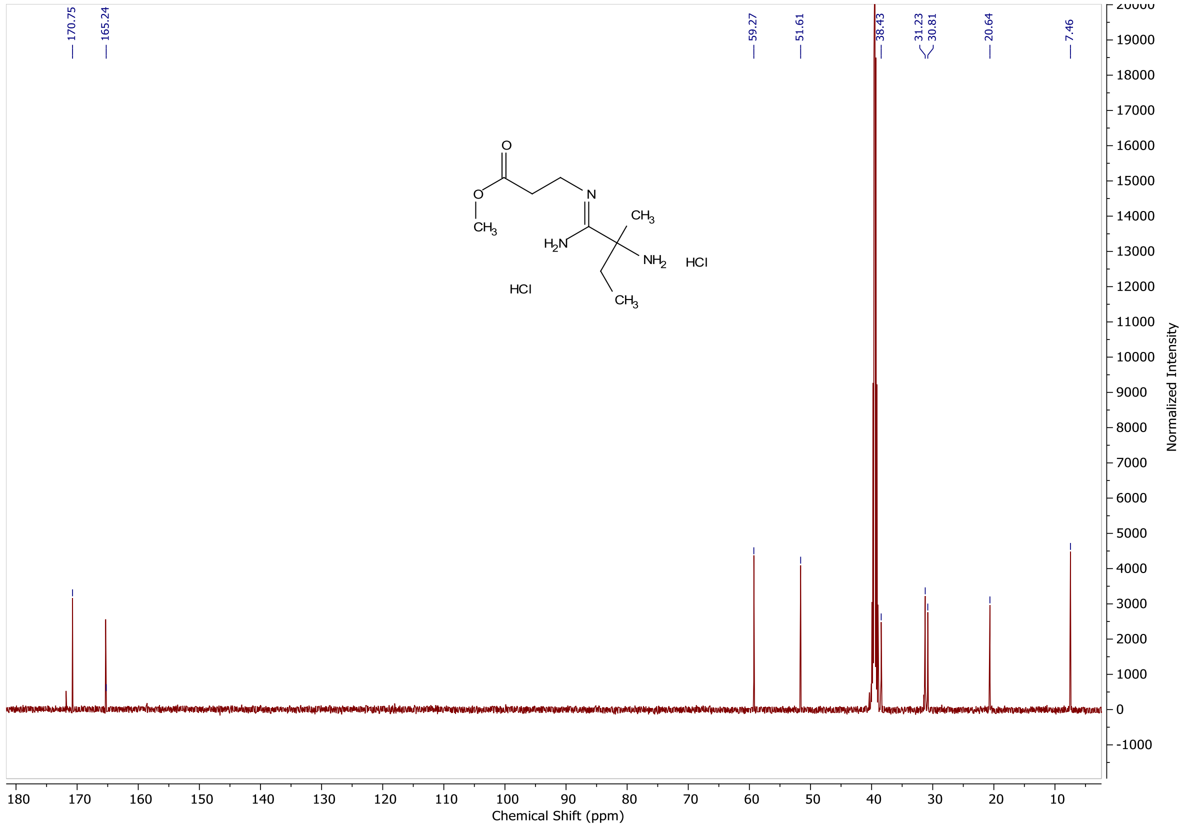

MASS SPECTRA OF 3, 5, 6, 8, 10A-K, 11 AND 12

MASS SPECTRUM 3

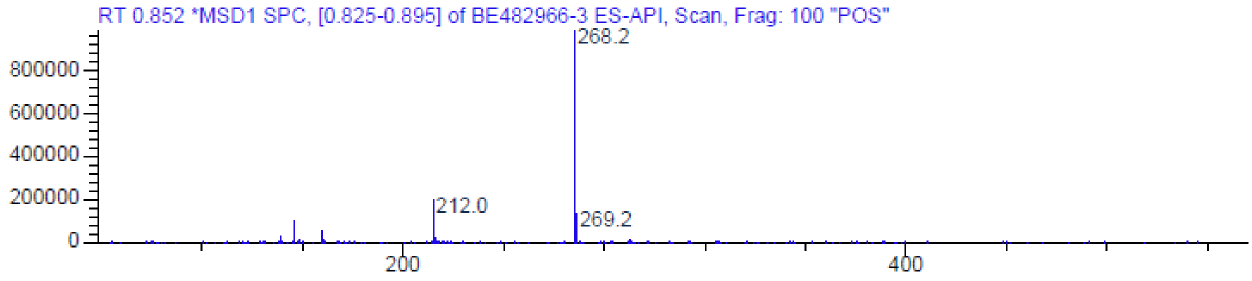

MASS SPECTRUM 5

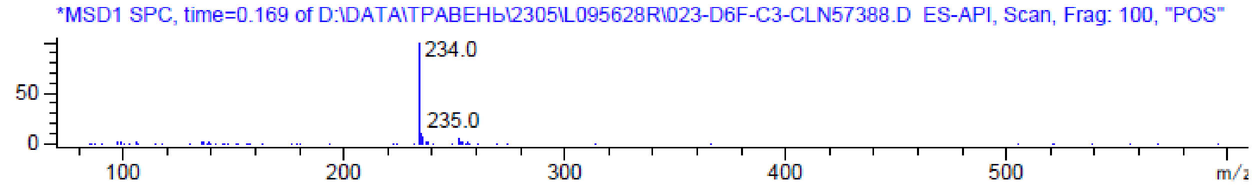

MASS SPECTRUM 6

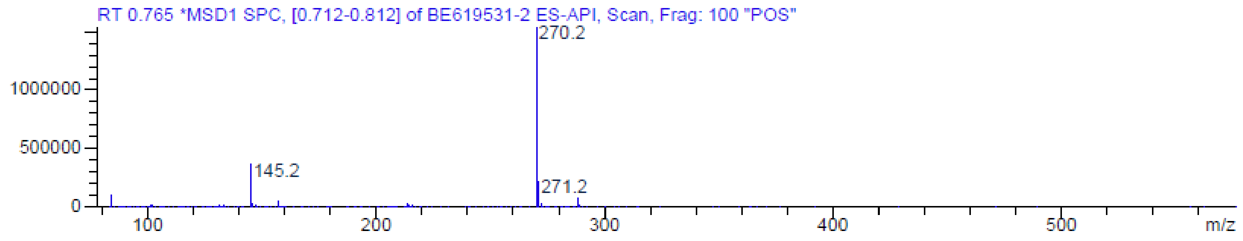

MASS SPECTRUM 8

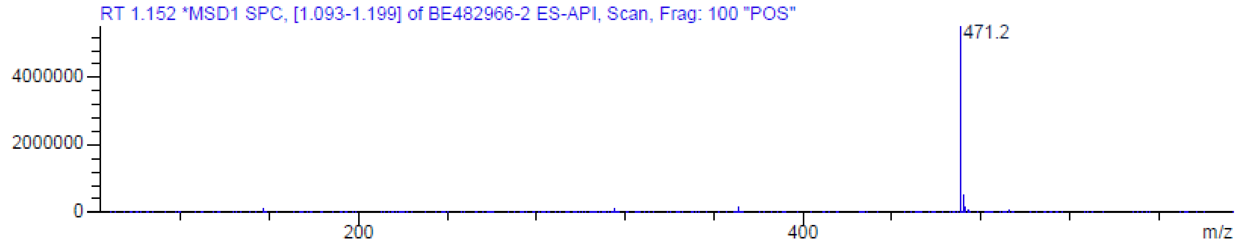

MASS SPECTRUM 10A

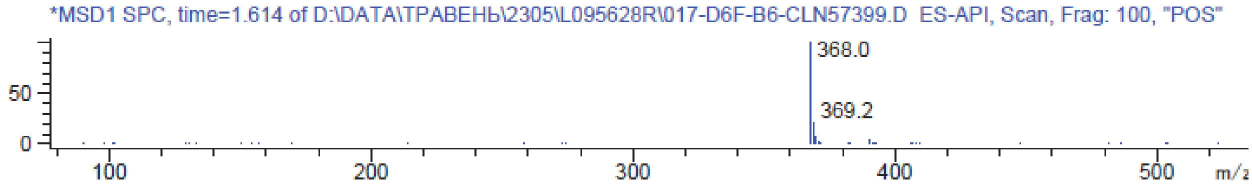

MASS SPECTRUM 10B

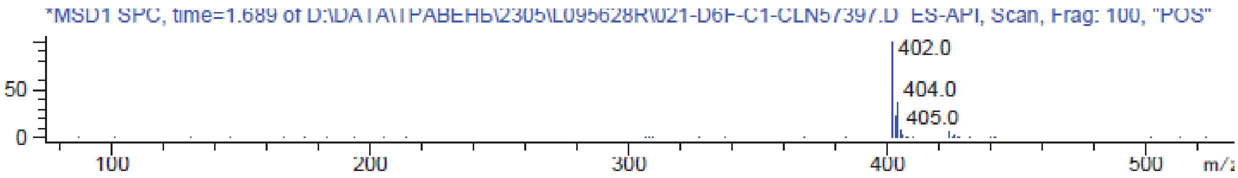

MASS SPECTRUM 10C

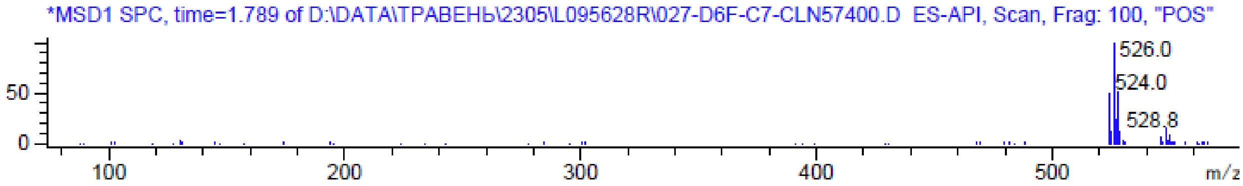

MASS SPECTRUM 10D

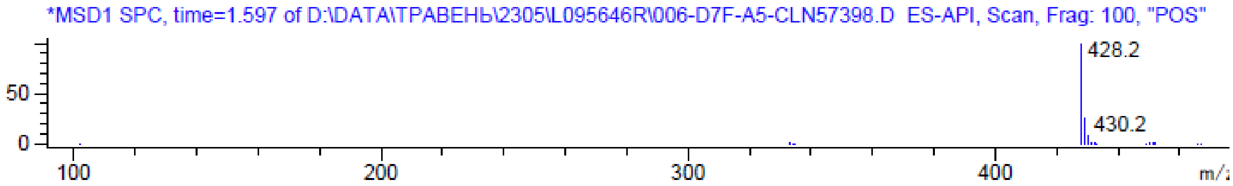

MASS SPECTRUM 10E

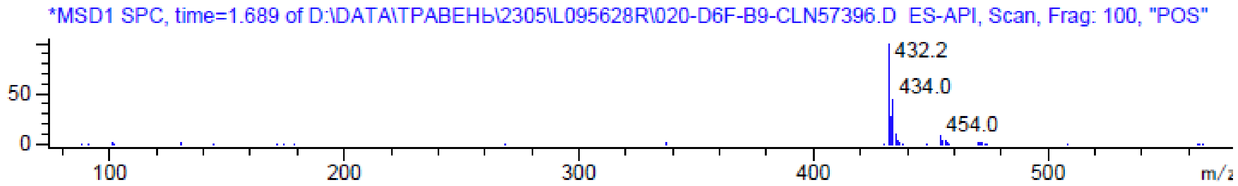

MASS SPECTRUM 10F

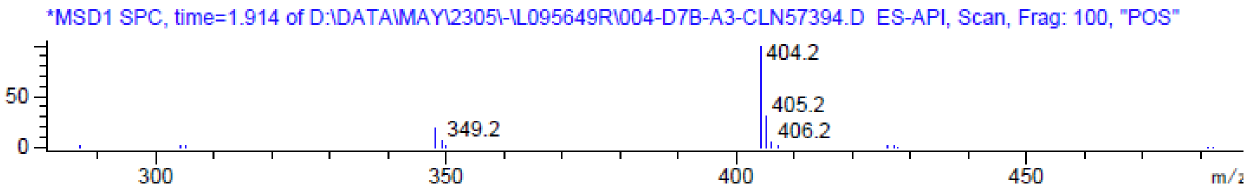

MASS SPECTRUM 10G

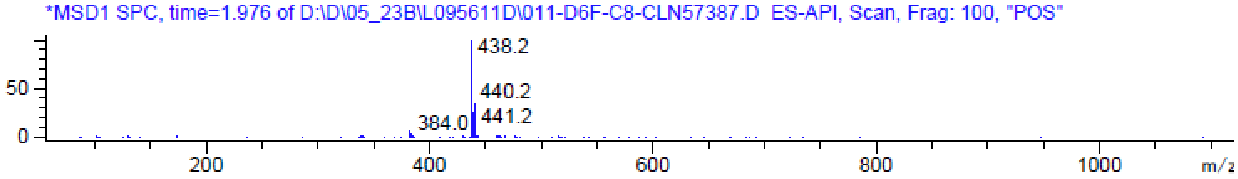

MASS SPECTRUM 10H

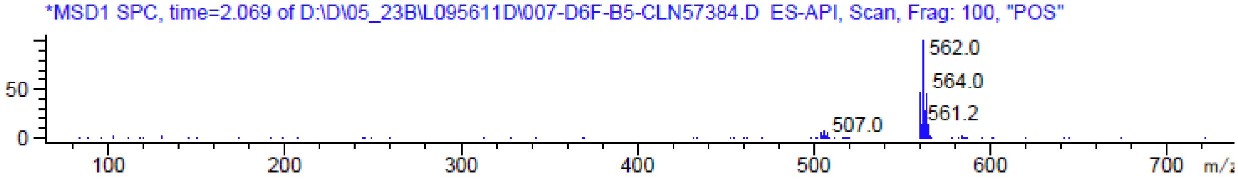

MASS SPECTRUM 10I

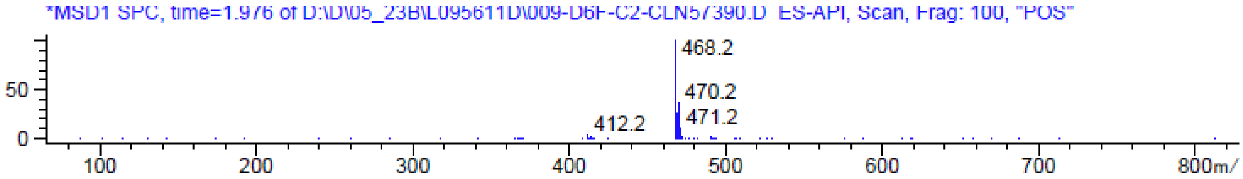

MASS SPECTRUM 11

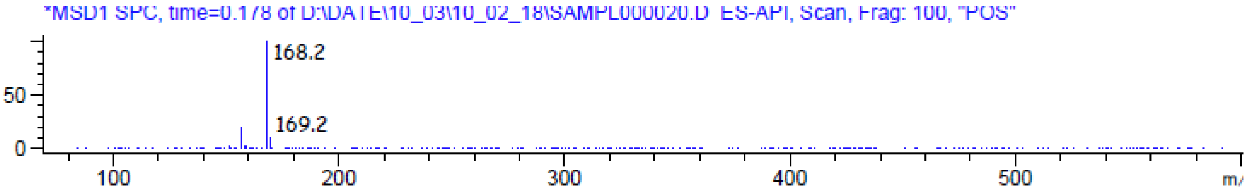

MASS SPECTRUM 12

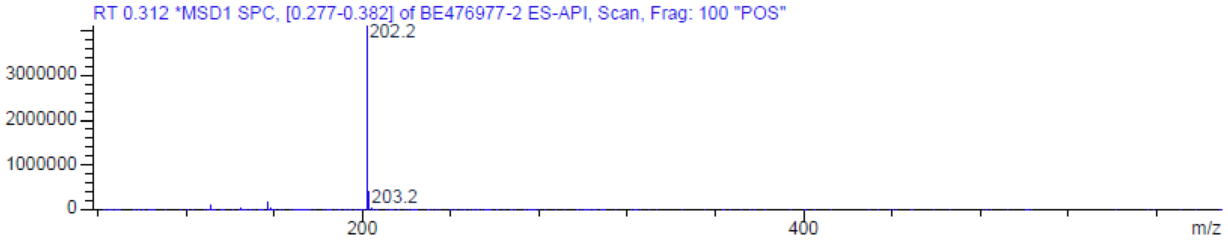

REFERENCES

[1] Maira S.-M., Pecchi S., Huang A., Burger M., Knapp M., Sterker D., Schnell C., Guthy D., Nagel T., Wiesmann M., Brachmann S., Fritsch C., Dorsch M., Chène P., Shoemaker K., De Pover A., Menezes D., Martiny-Baron G., Fabbro D., Wilson C. J., Schlegel R., Hofmann F., García-Echeverría C., Sellers W. R., Voliva C. F. Identification and characterization of nvp-bkm120, an orally available pan-class i pi3-kinase inhibitor. *Mol. Cancer Ther.* **2012**, *11* (2), 317-328. doi: 10.1158/1535-7163.MCT-11-0474

[2] Bohnacker T., Prota A. E., Beaufils F., Burke J. E., Melone A., Inglis A. J., Rageot D., Sele A. M., Cmiljanovic V., Cmiljanovic N., Bargsten K., Aher A., Akhmanova A., Díaz J. F., Fabbro D., Zvelebil M., Williams R. L., Steinmetz M. O., Wymann M. P. Deconvolution of buparlisib's mechanism of action defines specific pi3k and tubulin inhibitors for therapeutic intervention. *Nat. Commun.* **2017**, *8* (1), 14683. doi: 10.1038/ncomms14683
